# Venom-Derived Enzyme Inhibitors as Anticancer Agents: Structure–Activity Relationships, Molecular Targets and Mechanistic Insights

**DOI:** 10.3390/molecules31132398

**Published:** 2026-07-07

**Authors:** Ayorinde Victor Ogundele, Geetmani Singh Nongthombam, Adanna D. Nwagu, Héctor Hernán Silva, Oluwatoyin Adenike Fabiyi

**Affiliations:** 1 Departamento de Ciencias Basicas, Facultad de Medicina, Universidad de La Frontera, Temuco 4780000, Chile; hector.silva@ufrontera.cl; 2 Department of Chemistry (RSAPS), The Assam Royal Global University, Guwahati 781035, Assam, India; 3 Department of Chemistry, Universidad Tecnica Federico Santa Maria, Valparaíso 2340000, Chile; 4 Department of Crop Protection, Faculty of Agriculture, University of Ilorin, Ilorin 240003, Nigeria

**Keywords:** animal venoms, venom-derived enzyme inhibitors, anticancer agents, structure-activity relationships, molecular targets, matrix metalloproteinases, phospholipase A_2_

## Abstract

Animal venoms represent an extraordinary, yet largely untapped, biochemical reservoir for oncological drug discovery. This review provides a comprehensive analysis of venom-derived enzyme inhibitors as emerging anticancer agents, emphasizing their chemical diversity, structure–activity relationships (SAR), molecular targets, and mechanistic pathways. Venom-derived peptides and proteins exhibit exceptional binding affinity and structural rigidity, characteristics frequently enforced by conserved disulfide networks. This specific architecture allows them to selectively modulate critical cancer-associated enzymes, including matrix metalloproteinases, phospholipases A_2_, serine proteases, and kinases. Inhibiting these highly specific targets successfully disrupts tumour angiogenesis, extracellular matrix remodelling, and metastatic dissemination, while simultaneously inducing apoptosis through unique pathways such as reactive oxygen species generation. Modern computational approaches, encompassing deep learning algorithms, molecular docking, and molecular dynamics simulations, are substantially accelerating and transforming the discovery pipeline by rapidly mapping intricate peptide–receptor interactions and guiding rational drug design. Translating these potent molecules into clinical therapeutics remains heavily challenged by pharmacokinetic instability, rapid proteolytic degradation, and systemic toxicity. The integration of computationally optimized scaffolds with advanced targeted delivery platforms, such as nanocarriers and liposomal encapsulation, offers a highly viable strategy to overcome these barriers, ultimately paving the way for next-generation, venom-inspired cancer therapies.

## 1. Introduction

Cancer remains a leading global health challenge, accounting for approximately one in six deaths worldwide [1]. Despite significant advances in diagnosis and treatment, conventional therapeutic approaches such as chemotherapy, radiotherapy, and targeted therapies are often limited by systemic toxicity, off-target effects, and the emergence of drug resistance [2]. These limitations not only compromise therapeutic efficacy but also adversely affect patient quality of life. Consequently, there is a growing demand for alternative or complementary strategies that combine high selectivity with reduced toxicity, thereby improving clinical outcomes.

Natural products have historically played a central role in anticancer drug discovery, providing structurally diverse and biologically active compounds with unique mechanisms of action. Many clinically used anticancer agents are either directly derived from natural sources or inspired by natural scaffolds [3,4,5]. In this context, animal venoms have emerged as a particularly promising yet underexplored reservoir of bioactive molecules. Traditionally regarded solely as toxic secretions, venoms are now recognized as complex biochemical libraries containing a wide array of pharmacologically active components with high potency and specificity [6]. The successful translation of venom-based compounds into clinically proven therapies highlights the pharmaceutical utility of venoms from animals. A key example is captopril, an angiotensin-converting enzyme (ACE) inhibitor based on peptides isolated from the venom of the *Bothrops jararaca* snake. While captopril was discovered and approved as a drug for the treatment of high blood pressure and not cancer, its approval has provided a vital proof of principle for the successful translation of venom-based active compounds to pharmaceutical agents [7].

Venoms are intricate mixtures comprising peptides, proteins, enzymes, and low-molecular-weight organic compounds, alongside inorganic salts and other small biomolecules [8]. These components include neurotoxins, protease inhibitors, phospholipases, metalloproteinases, and various bioactive peptides, many of which exhibit strong biological activities [9]. Notably, venom-derived molecules often display remarkable selectivity toward specific molecular targets, including receptors, ion channels, and enzymes. Although millions of venom-derived compounds are believed to exist, only a small fraction has been characterized to date, largely due to challenges associated with isolation, purification, and limited natural abundance. However, recent advances in venomics integrating proteomics, transcriptomics, genomics, and computational approaches have significantly accelerated the identification and functional characterization of these compounds, opening new avenues for drug discovery [10].

Among the various mechanisms through which venom-derived compounds exert anticancer effects, enzyme inhibition has attracted considerable attention. Enzymes such as matrix metalloproteinases, phospholipases, kinases, and other regulatory proteins play critical roles in tumour progression, angiogenesis, invasion, and metastasis [11]. Targeting these enzymes offers a strategic approach to disrupt key pathways involved in cancer development. Venom-derived enzyme inhibitors are particularly appealing due to their high binding affinity, structural diversity, and ability to modulate specific biochemical pathways. In addition, many of these compounds have demonstrated selective cytotoxicity toward cancer cells, inducing apoptosis, inhibiting proliferation, and suppressing metastatic potential while sparing normal cells [12].

This increasing attention on venom-based therapies is supported by preclinical evidence demonstrating anticancer activity across multiple experimental systems. Venom peptides, proteins, and enzymes have exhibited cytotoxic, anti-proliferative, anti-metastatic, and pro-apoptotic effects in in vitro models of leukaemia, glioma, and lung, breast, prostate, and pancreatic cancers. However, most available evidence remains limited to in vitro investigations, with comparatively few candidates progressing into in vivo validation and only limited clinical translation to date [13]. Mechanistically, venom-derived compounds exert their biological effects through diverse pathways, including modulation of ion channels, activation of apoptotic signalling cascades, and inhibition or regulation of enzymes involved in tumour invasion, extracellular matrix remodelling, angiogenesis, and metastatic dissemination [14]. Given this mechanistic diversity, the present review specifically focuses on venom-derived molecules that either (i) directly inhibit cancer-relevant enzymes, (ii) modulate enzyme-regulated signalling pathways associated with tumour progression, or (iii) provide structurally informative scaffolds for the development of enzyme-targeted anticancer agents. Venom components whose dominant mechanisms involve membrane disruption, ion channel modulation, or broader cytotoxic activity are discussed only where they provide mechanistic context or demonstrate functional convergence with enzyme-mediated anticancer effects. Accordingly, this review is not intended to provide exhaustive coverage of all venom-derived anticancer compounds but instead emphasises molecules with established or emerging relevance to enzymatic targets in oncology.

Within this framework, the review provides a focused analysis of venom-derived enzyme inhibitors as emerging anticancer agents, with emphasis on their chemical diversity, structure–activity relationships, molecular targets, and mechanisms of action. Particular attention is given to the integration of computational approaches, including molecular docking and related in silico methodologies, to illustrate their contribution to target identification, binding analysis, and rational drug development. By consolidating current evidence and identifying important knowledge gaps, this review aims to provide a critical overview of the opportunities and limitations associated with translating venom-derived enzyme modulators into future oncology applications, while highlighting areas requiring further mechanistic and translational investigation.

### 1.1. Bibliometric Overview of Venom-Derived Enzyme Inhibitors in Cancer Research

A quantitative bibliometric investigation aimed to evaluate the historical trajectory and intensification of scientific interest in venom-derived enzyme inhibitors in the framework of oncological research was performed using Google Scholar database (28 February 2026) [15]. Only publications published within 2010–2026 period were included in the analysis. In order to ensure the precision of the searches and to exclude irrelevant entries, the Google Scholar advanced search options were used along with the title-restricted retrievals. The search strategy included the compulsory presence of the term “venom” in the “with all of the words” field, and any combination of at least one oncology-related and/or inhibition-related descriptors in the “with at least one of the words” field, which were as follows: inhibitor, inhibitors, cancer, tumour, tumour, oncology, anticancer. The surveyed material included only English-language scholarly original articles, review papers and book chapters. The following records were excluded: conference abstracts, editorials and corrections. Number of publications released in each year was counted for evaluation of research activity dynamics and identification of critical periods in development of the field. Bibliometric characteristics illustrate stable continuous long-term growth of venom-associated oncological research activity, although not always linear. The number of publications grew from 31 publications in 2010 to 104 publications in 2024, which is about three-and-a-half fold increase in the considered time period. Initial growth occurred within the 2010–2013 time period (31–85 publications), which can be explained by increasing interest to animal venoms as the source of pharmacologically active substances. After relatively stable changes in 2014–2019, the growth of research activity intensified starting from 2020. Since then, the annual production of publications remained consistently higher than 85 per year. It probably relates to the advances in technologies of venomics and omics platform, to increased interest in the mechanism-oriented anti-cancer screening and incorporation of computational methods in toxicology. Maximum of publication activity was registered in 2024 (104 publications) followed by stable productivity during 2025 (95 publications). Lower productivity in 2026 (47 publications) should not be treated as the decrease in research activity because the full-year data for 2026 is not available since the data collecting finished in February 2026.

**Figure 1:**
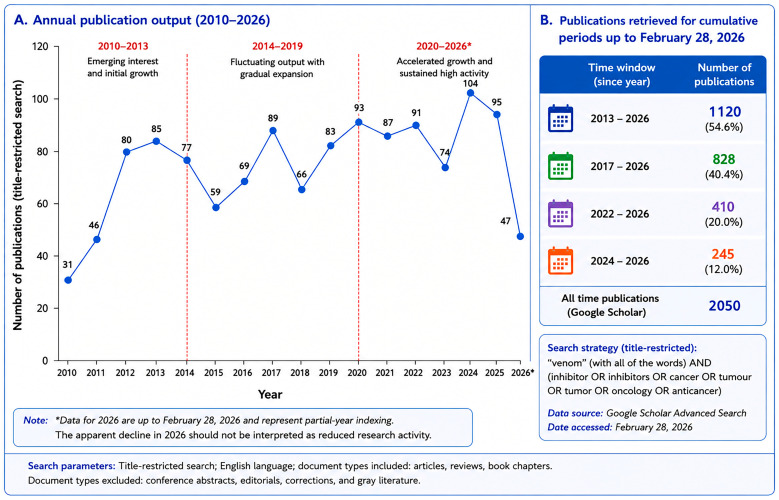
Publication trends in venom-derived enzyme inhibitor research in oncology (2010–2026).

The temporal aggregation confirms increasing intensity of research in recent years. About 55% of the obtained publications have been released starting from 2017 (828 publications), while about 20% of the publications were generated during the most recent period of 2024–2026 (245 publications). It indicates the ongoing increase of interests in venomous oncology research. The bibliometric picture shows gradual transition from the exploratory approach to cytotoxicity of venoms toward more mechanistic studies focused on enzyme modulation and molecular targeting. It corroborates the chosen thematic organization and emphasizes the growing opportunities of selective venom-derived enzyme inhibitor discoveries for anticancer applications. The quantitative publication trend supporting this analysis is summarized in Figure 1.

### 1.2. Literature Search Strategy and Review Methodology

The preparation of this review followed a structured literature identification and selection approach guided by principles of transparent evidence synthesis and informed by the PRISMA framework to enhance methodological consistency and reproducibility. Because this manuscript is a narrative and mechanistic review rather than a formal systematic review or meta-analysis, PRISMA procedures were applied as guidance for literature identification, screening, and reporting rather than as a strict methodological requirement.

A comprehensive literature search was conducted using multiple electronic databases, including PubMed, Scopus, Web of Science Core Collection (WoSCC), Google Scholar, and Mendeley-supported reference management and screening workflows. Additional studies were identified through manual screening of reference lists from selected articles, relevant review papers, and leading journals in toxinology, oncology, medicinal chemistry, pharmacology, natural products research, and translational medicine.

The literature search covered publications from January 2010 to February 2026 for the overall review. For Section 5 (Computational Approaches and Mechanistic Insights) and 6 (Preclinical and Clinical Evidence), particular emphasis was placed on the literature published between 2014 and 2025 to capture contemporary advances in computational venomics, molecular modeling, translational oncology, and clinical development, while seminal earlier studies were retained where necessary to provide historical and mechanistic context.

Search terms were developed iteratively and combined using Boolean operators. Representative search combinations included terms related to venom-derived compounds (“venom”, “animal venom”, “snake venom”, “scorpion venom”, “spider venom”, “marine venom”, “toxin”, “peptide”, “phospholipase A_2_”, “disintegrin”, “L-amino acid oxidase”), enzyme-related mechanisms (“enzyme inhibitor”, “enzyme modulation”, “metalloproteinase”, “serine protease”, “enzyme target”), computational methodologies (“molecular docking”, “molecular dynamics simulation”, “virtual screening”, “machine learning”, “artificial intelligence”, “structure prediction”), and oncology-related descriptors (“cancer”, “tumour”, “oncology”, “anticancer”, “apoptosis”, “cell cycle arrest”, “enzyme inhibition”, “in vitro”, “in vivo”, “xenograft”, “pharmacokinetics”, “ADMET”, and “clinical trials”).

Eligibility criteria included the following: (i) peer-reviewed original research articles, review articles, and clinical studies; (ii) studies investigating venom-derived peptides, proteins, enzymes, or enzyme inhibitors with demonstrated or proposed anticancer relevance; (iii) reports containing structural, mechanistic, structure–activity relationship (SAR), computational, preclinical, translational, or clinical information; and (iv) publications written in English. Exclusion criteria comprised the following: (i) conference abstracts, editorials, corrections, dissertations, patents, and non-peer-reviewed literature; (ii) studies lacking sufficient mechanistic, structural, or experimental detail; (iii) reports focused exclusively on antivenom development, ecological toxinology, or envenomation without relevance to cancer biology; and (iv) duplicate records identified across databases.

Study selection was performed in sequential stages involving title screening, abstract assessment, and full-text evaluation. Articles meeting predefined eligibility criteria were retained for qualitative synthesis. For Section 5 and Section 6 specifically, 184 records were initially identified; following screening and eligibility assessment, 62 records were excluded and 122 studies were retained for detailed analysis. Extracted information included venom source, toxin class, chemical structure, molecular targets, enzyme systems, computational methodologies, pharmacokinetic characteristics, mechanistic outcomes, preclinical efficacy, and clinical development status.

The selected literature was subsequently organized and synthesized according to venom origin, chemical diversity, enzyme targets, structure–activity relationships, mechanisms of action, computational approaches, translational progress, and current challenges limiting clinical implementation. This structured methodology was adopted to improve transparency and provide a reproducible framework for evaluating the evolving landscape of venom-derived enzyme inhibitors in cancer research.

## 2. Overview of Venoms as a Source of Bioactive Compounds

Venom secretion is a highly specialized biochemical system that evolved to regulate essential physiological processes in prey and predators through selective interactions with defined molecular targets. Animal venoms comprise structurally diverse bioactive constituents, including peptides, proteins, enzymes, enzyme inhibitors, and low-molecular-weight compounds, many of which display high affinity, target selectivity, and potent biological activity. These molecules possess favorable physicochemical characteristics such as conformational stability, receptor specificity, proteolytic resistance, and the ability to interact with complex biological interfaces, making them attractive templates for anticancer drug discovery [4,14].

However, despite the broad anticancer potential reported for venom-derived molecules, only a subset exerts its activity through direct inhibition of cancer-associated enzymes or through modulation of enzyme-regulated signaling networks that govern tumor growth, invasion, angiogenesis, metastasis, and apoptotic responses. Numerous venom compounds additionally exhibit membrane-disruptive, ion-channel-modulating, or broader cytotoxic effects that contribute to anticancer activity but do not represent classical enzyme inhibition mechanisms. Accordingly, this section focuses primarily on venom-derived compounds with demonstrated enzymatic relevance, whereas non-enzyme-centered mechanisms are discussed only where they provide biological context or illustrate mechanistic convergence with enzyme-mediated anticancer pathways.

The following sections therefore emphasize venom-derived compounds and molecular classes that either directly target enzymes implicated in cancer progression or provide mechanistically informative scaffolds for the development of future enzyme-directed anticancer therapeutics. Pharmacologically, these molecules are able to affect tumor-related processes using several strategies, such as enzyme inhibition, interference with signal transduction pathways, membrane destabilization, redox modulation, and blocking cell-matrix interactions. The presence of structural motifs, such as disulfide-rich framework, amphipathy, and binding sites, helps venom-related compounds selectively interact with the enzymes and related protein complexes involved in cancerogenesis [8] as presented in Table 1. These facts made researchers focus on the use of venom molecules as the templates for new generation anticancer drugs. This section will review the chemical diversity of venom molecules and structural aspects that influence their ability to target enzymes and exert the anticancer effects.

Venoms are produced by a wide range of organisms, including insects (ants, bees, wasps, beetles), arachnids (scorpions and spiders), vertebrates (notably snakes), and other invertebrates such as myriapods. This phylogenetic diversity translates into substantial chemical heterogeneity. Venoms typically comprise complex mixtures of low-molecular-weight metabolites, linear and disulfide-rich peptides, globular proteins, and catalytically active enzymes, alongside inorganic ions [16,17]. Structurally, many venom peptides fall into defined classes such as α-helical amphipathic peptides or cysteine-rich frameworks (e.g., inhibitor cystine knot motifs), which confer high stability and target specificity. Enzymatic constituents including phospholipases A_2_, metalloproteinases, serine proteases, and oxidases are particularly relevant to cancer biology due to their capacity to regulate membrane dynamics, extracellular matrix (ECM) remodeling, and intracellular signaling cascades [18].

Insect venoms provide illustrative examples of chemically distinct bioactive compounds with anticancer relevance. Alkaloidal components such as solenopsin A from fire ant venom act as inhibitors of phosphatidylinositol-3-kinase (PI3K), suppressing angiogenesis and tumor-associated vascularization [19]. Peptide-rich bee venom contains melittin, a prototypical amphipathic α-helical peptide that integrates into lipid bilayers, causing membrane destabilization and lytic cell death [20]. Despite the lack of designation of melittin as an enzyme inhibitor, the anticancer activities of melittin include more than just membrane disruption; the protein affects signaling pathways inside the cell. In particular, it was found that melittin inhibits NF-κB signaling, induces death receptor-mediated apoptosis, and creates endoplasmic reticulum stress, which results in apoptosis and ferroptosis of cancer cells [21]. These effects are often enhanced by enzymatic partners such as phospholipase A_2_, highlighting the cooperative nature of venom components.

Blister beetle toxins further exemplify enzyme-targeted mechanisms. Cantharidin, a bicyclic terpenoid, is a potent inhibitor of serine/threonine protein phosphatases PP1 and PP2A, leading to the dysregulation of phosphorylation-dependent signaling, DNA damage, and apoptotic cell death [22]. Its derivatives have demonstrated activity across multiple cancer types and are particularly notable for their capacity to suppress metastasis via inhibition of matrix metalloproteinases [23,24]. In addition, peptides such as cecropins from caterpillars exhibit selective anticancer effects through membrane disruption, inducing pore formation in cancer cells and inhibiting their proliferation without affecting normal cells [25].

Venoms from arachnids and snakes show significant variations in molecular composition and determine the specific mechanisms of their antitumor activity. Venoms of scorpions and spiders contain numerous small disulfide-rich peptides specifically designed for interaction with membrane-bound targets (ion channels, receptors). These peptides do not directly inhibit enzymes but influence enzyme-related signaling cascades due to their ability to affect ion homeostasis and intracellular signal transduction. Most scorpion venom peptides act as inhibitors of voltage-dependent sodium, potassium, and chloride channels, producing subsequent effects on calcium signaling, kinase activation, mitochondrial function, and apoptotic signaling [26]. Such effects may have some impact on the progression of tumors due to the involvement of proliferation, migration, and survival into these processes. One of the most studied peptides of this type, chlorotoxin (CTX), demonstrates selective binding to tumor membrane targets and potential anti-invasion properties (Section 4). Another group of venoms, spider venoms, includes a number of peptides able to impair mitochondrial function, activate caspases, produce oxidative stress, and cause cell cycle alterations resulting in programmed cell death [27].

As opposed to arachnid venoms, snake venoms are rich in catalytically active toxins and modulators of endogenous enzymes, making them particularly relevant for this review. The main functional groups include phospholipases A_2_, metalloproteinases, serine proteinases, disintegrins, and L-amino acid oxidases (LAAO). In turn, phospholipases A_2_ alter the composition of membrane lipids and signaling, while LAAO cause oxidative stress through production of hydrogen peroxide, metalloproteinases alter extracellular matrix and interactions with tumor microenvironment [28]. Other components of venoms may modulate signaling cascades responsible for apoptosis and cell survival (NF-κB, STAT3), as well as serve as delivery vectors penetrating into tumor cells [29]. From these observations, one can conclude that venom-based antitumor activity is not provided by the same mechanism but rather appears to be a result of an interaction of molecular structure and target specificity. Disulfide-rich peptides generally demonstrate preferences toward the modulation of receptors and ion channels, while enzyme toxins prefer catalytic and microenvironmental processes affecting tumor progression [30].

Hymenopteran venoms (e.g., wasps) and myriapod toxins contribute additional structurally unique compounds. Peptides such as mastoparan and polybia-MPI short, amphipathic α-helical peptides exert cytotoxic effects through membrane disruption and mitochondrial permeabilization, coupled with activation of caspase-dependent apoptosis [31]. Importantly, these peptides are amenable to structural modification and conjugation with targeting ligands, facilitating the development of tumor-selective delivery systems. Similarly, centipede-derived compounds have been shown to inhibit key signaling axes, including PI3K/Akt and MAPK pathways, through the modulation of upstream enzymatic regulators, resulting in cell cycle arrest and apoptosis [32].

**Table 1:** Representative venom-derived compounds, structural classes, molecular targets, and anticancer activities.

Source	Compound	Representative Structure	Structure Classes	Molecular Targets	Mechanism of Actions	Mechanistic Classification	Cancer Context	Ref.
*Solenopsis invicta* (Fire ant)	Solenopsin A	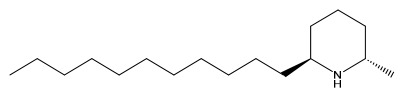	Alkaloid	PI3K/Akt pathway	Anti-angiogenic signaling	Enzyme pathway modulator	Breast	[19]
*Apis mellifera* (Bee)	Melittin	Peptide	Amphipathic peptide	NF-κB, PLA_2_-associated pathways	Membrane disruption; apoptosis	Context-only	Leukemia, lung, breast, prostate	[20,21]
*Mylabris* spp. (Blister beetle)	Cantharidin	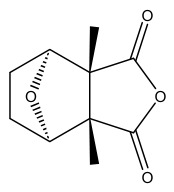	Terpenoid	PP1, PP2A	Phosphatase inhibition; apoptosis	Direct enzyme inhibitor	Liver, breast	[22,23,24]
*Hyalophora cecropia* (Caterpillar)	Cecropin A/B	Peptide	Linear cationic peptide	Membrane	Membrane permeabilization	Context-only	Lung, colon	[25]
*Leiurus quinquestriatus* (Scorpion)	Chlorotoxin	Peptide	Disulfide peptide	MMP-2-related pathways	Anti-invasion; anti-metastatic	Enzyme pathway modulator	Glioma	[26]
*Buthus martensii* (Scorpion)	Gonearrestide	Peptide	Peptide	Cell-cycle pathways	Cell-cycle arrest	Enzyme pathway modulator	Multiple	[27]
*Macrothele raveni* (Spider)	Venom peptides	Peptides	Peptides	Caspase pathways	Apoptosis; signaling modulation	Enzyme pathway modulator	Multiple	[28]
*Lycosa vittata* (Spider)	Venom extract	Peptides	Peptide mixture	Mitochondrial apoptotic pathway	Broad antiproliferative effects	Context-only	Multiple	[29]
*Ophiophagus hannah* (King cobra)	L-amino acid oxidase (LAAO)	Protein scaffold	Enzyme (flavoprotein)	Redox pathways	ROS-mediated apoptosis	Enzyme-derived anticancer effector	Breast, lung cancer	[30]
*Vipera* spp. (Viper)	Metalloproteinases	Protein scaffold	Enzyme	ECM	ECM remodeling; invasion control	Direct enzyme inhibitor	Solid tumors	[18]
*Crotalus durissus terrificus* (Rattlesnake)	Crotamine	Peptide	Cell-penetrating peptide	Intracellular organelles	Intracellular targeting	Context-only	Melanoma	[32]
*Polybia paulista* (Wasp)	Polybia-MPI	Peptide	Amphipathic peptide	Membrane	Selective membrane lysis	Context-only	Prostate, bladder,	[33]
*Polistes jadwigae* (Wasp)	Mastoparan	Peptide	Amphipathic peptide	Mitochondrial signaling	Mitochondrial apoptosis	Context-only	Breast	[33]
*Scolopendra subspinipes* (Centipede)	Venom extract	Protein scaffold	Peptides/proteins	PI3K/Akt, MAPK pathways	Broad anti-proliferative effects	Context-only	Melanoma, cervical cancer	[34,35]

**Figure 2:**
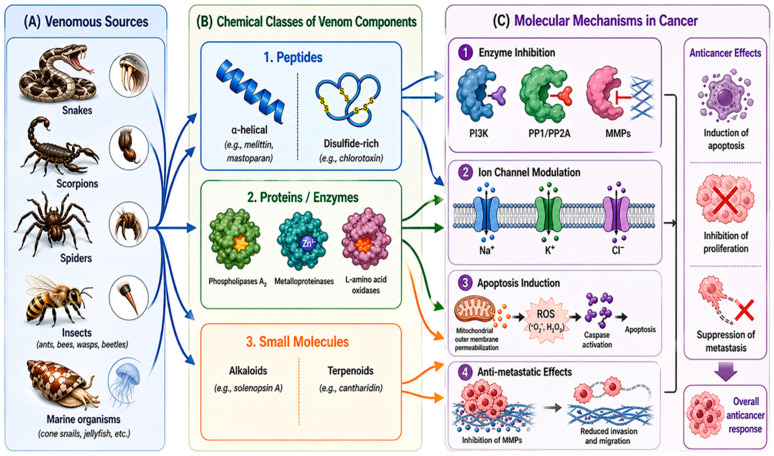
Schematic representation of the diversity of venomous organisms (**A**), the major chemical classes of venom-derived compounds (**B**), and their principal molecular mechanisms of anticancer action (**C**). Venom-derived peptides, proteins/enzymes, and small molecules interact with key molecular targets, including enzymes, ion channels, and intracellular signaling pathways, leading to apoptosis, inhibition of proliferation, and suppression of metastasis.

Despite the remarkable diversity and therapeutic promise of venom-derived compounds, their full potential remains largely untapped. It is estimated that only a minute fraction of venom components has been identified and characterized, primarily due to challenges associated with low yield, structural complexity, and difficulties in isolation and purification [33]. However, recent advances in analytical and molecular technologies have significantly accelerated venom research. The advent of venomics an integrated framework combining proteomics, transcriptomics, and metabolomics has enabled comprehensive profiling of venom composition. Modern workflows typically involve multidimensional chromatographic or electrophoretic separation, followed by enzymatic digestion and high-resolution mass spectrometric analysis using MALDI or electrospray ionization coupled with tandem MS. These approaches allow for the precise identification, sequencing, and relative quantification of venom peptides and proteins. Integration with transcriptomic datasets from venom glands enhances annotation accuracy and facilitates the discovery of novel compounds, while computational tools support structural modelling, enzyme–ligand interaction analysis, and target prediction [7]. In other words, these advances have accelerated the identification of venom-derived molecules with therapeutic relevance (Figure 2).

## 3. Enzymes as Therapeutic Targets in Cancer

An overview of the enzymatic landscape involved in the growth and spread of cancer is crucial in placing the therapeutic value of venom enzymes inhibitors to be elaborated on in subsequent sections. Because venoms act through interactions with specific biological targets, including catalytic enzymes, enzyme-mediated pathways, and enzyme-regulated signaling pathways, it is important first to identify the major groups of enzymes involved in tumor formation and currently represent validated targets in oncology. Cancer results from aberrations in biochemical and signaling pathways, where enzymes control cell proliferation, survival, angiogenesis, invasion, metastasis, and therapy resistance [36]. In addition, aberrant enzyme activity or expression directly participates in the pathogenesis of tumors by controlling ECM degradation, signal transduction, DNA synthesis, epigenetic modifications, and metabolism [37]. Thus, enzymes have become some of the most clinically validated therapeutic targets in oncology, with many enzyme-targeted therapies being used in routine cancer treatment. However, not all groups of enzymes equally participate in cancer pathogenesis and share the same therapeutic potential. The groups of enzymes relevant to cancer biology include proteases, kinases, topoisomerases, phospholipases, epigenetic enzymes, and nucleotide biosynthetic enzymes. All of these enzymes are responsible for tumor maintenance, and their activity can be altered pharmacologically. Mapping these enzyme families will allow us to identify the mechanisms through which venom molecules inhibit or regulate the enzyme activity or the activity of the enzyme-controlled pathways.

The therapeutic relevance of enzymes in cancer is attributed to several factors. Many cancer-associated enzymes are overexpressed, constitutively activated, or selectively enriched within tumor tissues, thereby providing opportunities for targeted intervention. In addition, the catalytic nature of enzymes enables substantial biological effects to be achieved even through partial inhibition. Their structurally defined active or allosteric sites further facilitate rational drug design and structure–activity optimization [38]. Importantly, venom-derived molecules have increasingly attracted attention as promising modulators of cancer-associated enzymes owing to their remarkable structural diversity, target affinity, and unique inhibitory mechanisms.

### 3.1. Major Enzyme Classes Targeted in Oncology

#### 3.1.1. Proteases

Proteases play essential roles in tumor progression through degradation of ECM components, activation of signaling molecules, and modulation of the tumor microenvironment. Enhanced proteolytic activity promotes cancer cell invasion, angiogenesis, metastasis, and epithelial–mesenchymal transition (EMT) [39]. Among the major protease families implicated in cancer are matrix metalloproteinases (MMPs), serine proteases, cysteine cathepsins, and aminopeptidases.

**(1)**  Matrix Metalloproteinases (MMPs)

MMPs are zinc-dependent endopeptidases responsible for the degradation of collagen, laminin, fibronectin, and other ECM constituents. Although physiologically involved in tissue remodeling and wound repair, dysregulated MMP activity contributes significantly to malignant progression [40]. MMP-2 and MMP-9 are particularly associated with aggressive tumor phenotypes and poor prognosis due to their ability to facilitate ECM degradation and promote angiogenesis through the release of matrix-bound growth factors such as vascular endothelial growth factor (VEGF) [41]. Early broad-spectrum MMP inhibitors demonstrated limited clinical success because of poor selectivity and dose-limiting toxicity. However, renewed interest has emerged through the development of isoform-selective inhibitors and protease-activated therapeutic systems. Venom-derived molecules have also demonstrated MMP-modulating properties. For example, BJ46A, a snake venom metalloproteinase inhibitor, suppresses melanoma and hepatocellular carcinoma invasion through inhibition of MMP-2 and MMP-9 activity [42]. In addition, snake venom peptide libraries have yielded bioactive compounds with anti-invasive and anti-angiogenic properties, highlighting venom-derived scaffolds as promising templates for MMP-targeted anticancer strategies [12].

**(2)**  Serine Proteases

One way of serine protease participation in oncogenic progression includes induction of proteolysis cascades, ECM remodeling and signaling pathway alteration controlling invasion, angiogenesis and metastasis. As far as the field of oncology is concerned, these enzymes are mainly considered to be targets for therapy with the focus being on their activity inhibition in order to control tumor progression without using these enzymes for therapeutic purposes. One of the best characterized examples of the latter is urokinase-type plasminogen activator (uPA) system. UPA acts as a protease that converts plasminogen to plasmin, which leads to degradation of ECM components and activation of latent MMPs contributing to tumor invasion and metastasis [43]. High levels of uPA and uPA receptor (uPAR) are correlated with poor prognosis in a number of cancers. Matriptase is another clinically relevant serine protease involved in tumor growth, invasion and metastatic progression. Hence, selective inhibition of uPA, matriptase and other serine proteases is a promising tool to combat metastasis [44].

As opposed to the aforementioned cancer-related serine proteases serving as the targets for therapy, there are several molecules found in venom that inhibit serine protease pathways and hence can be considered as potential candidates for anticancer scaffolds. Kunitz-type serine protease inhibitors isolated from snake venoms do not play the role of tumor-progression related proteases. On the contrary, they act as inhibitors of serine proteases via inhibitory interaction with the target enzymes and related signaling pathways. Such examples include PIMR from *Macrovipera razii* venom and PIVL from *Macrovipera lebetina* venom possessing anticancer-related activity like inhibition of angiogenesis, proteolysis and integrin signaling interference [45].

**(3)**  Cysteine Cathepsins

Cysteine cathepsins are lysosomal proteases that become aberrantly expressed and secreted during cancer progression. Cathepsin B is particularly important in tumor biology and has been implicated in glioma, breast, prostate, colorectal, and lung cancers. Elevated cathepsin activity promotes ECM degradation, angiogenesis, EMT, and activation of additional proteolytic enzymes [46]. The imbalance between cathepsins and their endogenous inhibitors, cystatins, favors tumor invasion and metastasis. Pharmacological inhibition of cathepsins has therefore emerged as a promising therapeutic strategy. Recent approaches include cathepsin-responsive drug delivery systems and tumor-selective prodrugs that exploit elevated cathepsin activity within the tumor microenvironment [47].

**(4)**  Aminopeptidase N (APN/CD13)

Aminopeptidase N (APN/CD13) is a metalloprotease which is overexpressed in many solid tumors and blood cancers and depends on zinc ions for its activity. The role of APN in angiogenesis, migration, invasion, and metastasis in cancer is well-established. Overexpression of APN has been found to correlate with an aggressive tumor phenotype and poor prognosis [48]. Thus, APN has become an attractive pharmacological target in oncology. One of the first APN inhibitors, bestatin (ubenimex), proved the hypothesis of a possibility to slow down tumor growth and angiogenesis through pharmacological inhibition of APN activity [49]. In addition to clinically studied inhibitors, there are some naturally occurring non-venom compounds which have shown modulatory effect on APN activity. For instance, curcumin, a plant polyphenol, has been found to inhibit APN activity and have anti-angiogenic properties; yet it is mentioned here only as an example demonstrating the possibility of APN modulation by natural compounds and should not be considered as a venom derivative. In addition to APN enzyme inhibition, another important property of APN, which is being used in developing cancer-targeted therapies, is its ability to act as a receptor for tumor-homing NGR peptides [50]. This opens the way for designing future anticancer drug delivery methods involving both NGR peptides and venom-derived inhibitors of APN.

#### 3.1.2. Kinases

Protein kinases regulate essential cellular processes through phosphorylation-mediated signal transduction. Dysregulated kinase activity is a hallmark of many cancers and contributes to uncontrolled proliferation, evasion of apoptosis, angiogenesis, metastasis, and therapeutic resistance. Consequently, kinases represent one of the most extensively targeted enzyme classes in oncology [51]. Clinically approved kinase inhibitors target numerous oncogenic pathways, including EGFR, BRAF, ALK, VEGFR, MET, PI3K, CDKs, and BTK. Mechanistically, kinase inhibitors may function as ATP-competitive inhibitors, allosteric modulators, or irreversible covalent inhibitors targeting nucleophilic residues within kinase active sites. Despite the clinical success of kinase-targeted therapies, acquired resistance remains a major challenge due to secondary mutations, pathway redundancy, and compensatory signaling activation [52]. This has stimulated the development of next-generation inhibitors, combinatorial therapeutic approaches, and targeted protein degradation technologies. Venom-derived molecules represent a promising source of novel kinase modulators. Snake venom disintegrins interfere with integrin-associated kinase signaling pathways involved in migration and survival, whereas several scorpion and bee venom peptides modulate receptor-mediated signaling cascades associated with proliferation and apoptosis [26]. The structural diversity of venom-derived peptides provides valuable scaffolds for the development of selective kinase-targeted therapeutics.

#### 3.1.3. Topoisomerases

DNA topoisomerases are essential enzymes that regulate DNA topology during replication, transcription, recombination, and chromatin remodeling. Topoisomerase I (TOP1) introduces transient single-strand DNA breaks to relieve torsional stress, whereas topoisomerase II (TOP2) generates transient double-strand breaks to resolve DNA entanglement and chromosome segregation [53]. Because rapidly proliferating cancer cells are highly dependent on these processes, topoisomerases remain among the most clinically validated enzyme targets in oncology. Clinically approved topoisomerase inhibitors; including camptothecin derivatives for example; irinotecan and topotecan and TOP2-directed agents like etoposide and doxorubicin act predominantly by stabilizing transient enzyme–DNA cleavage complexes, thereby converting physiological DNA processing events into cytotoxic DNA damage [54,55]. Despite their clinical success, these agents are limited by systemic toxicity, secondary malignancies, and acquired resistance mechanisms involving altered drug transport, enhanced DNA repair, and target adaptation [56].

In contrast to proteases and phospholipases, topoisomerases remain comparatively underrepresented among validated targets of venom-derived anticancer agents. Current evidence suggests that most venom-derived compounds associated with DNA damage exert indirect effects through oxidative stress, membrane perturbation, mitochondrial dysfunction, or activation of apoptotic pathways rather than through direct and selective inhibition of TOP1 or TOP2. For example, snake venom LAAOs generate reactive oxygen species capable of inducing DNA fragmentation and apoptosis, while some venom peptides influence cell cycle progression and DNA damage responses without confirmed topoisomerase binding [30].

Therefore, topoisomerases are discussed here not as established venom-sensitive enzyme targets, but as an important knowledge gap and future opportunity for venom-based drug discovery. The structural diversity and target selectivity of venom-derived peptides may provide opportunities to develop alternative DNA-processing modulators or tumor-selective delivery systems capable of overcoming the limitations of conventional topoisomerase-directed therapies.

#### 3.1.4. Phospholipases A_2_ (PLA_2_s)

PLA_2_ enzymes catalyse the hydrolysis of membrane phospholipids, generating free fatty acids and lysophospholipids that regulate inflammation, membrane dynamics, apoptosis, and cell signaling. Dysregulated PLA_2_ activity contributes to tumor progression through promotion of angiogenesis, inflammatory signaling, and EMT [57]. Secreted PLA_2_ isoforms have attracted significant interest as therapeutic targets due to their involvement in cancer-associated inflammatory pathways. Conversely, venom-derived PLA_2_s themselves possess potent anticancer properties. Snake venom PLA_2_s and bee venom PLA_2_s induce membrane destabilization, mitochondrial dysfunction, oxidative stress, apoptosis, autophagy, and cell cycle arrest in diverse cancer cell lines [58,59,60]. The anticancer effects of venom PLA_2_s are often enhanced by synergistic venom peptides such as melittin, which facilitates membrane permeabilization and increases phospholipid accessibility [61]. These observations highlight venom PLA_2_s not only as therapeutic candidates but also as valuable molecular models for understanding phospholipid-mediated cancer signaling.

#### 3.1.5. Epigenetic Enzymes

Epigenetic dysregulation is a defining feature of cancer and contributes to tumor initiation, progression, immune evasion, and therapeutic resistance. Enzymes that regulate chromatin organization and DNA methylation have therefore become important therapeutic targets in oncology. Although clinically established epigenetic therapies exist, their direct exploitation by venom-derived inhibitors remains limited, highlighting an emerging rather than mature area of venom-based anticancer research.

**(1)**  Histone Deacetylases (HDACs)

HDACs regulate chromatin accessibility and gene expression through removal of acetyl groups from lysine residues on histones and non-histone proteins. Aberrant HDAC activity promotes tumor progression by suppressing tumor suppressor genes, enhancing proliferation, impairing differentiation, and promoting resistance to apoptosis [62]. Consequently, HDAC inhibition has become an established anticancer strategy, particularly in haematological malignancies. Several HDAC inhibitors like vorinostat, belinostat, romidepsin, and panobinostat have demonstrated clinical utility and are increasingly investigated in combination regimens to improve efficacy in solid tumors [63]. However, well-characterized venom-derived molecules acting as direct and selective HDAC inhibitors have not yet been established. Nevertheless, emerging evidence suggests that certain venom components may indirectly influence epigenetic regulation through modulation of oxidative stress pathways, transcriptional responses, apoptosis signaling, and chromatin-associated processes [64]. These observations suggest that venom-derived scaffolds may represent future starting points for developing epigenetic modulators, although this concept remains largely exploratory and requires mechanistic validation.

**(2)**  DNA Methyltransferases (DNMTs)

DNMTs catalyse methylation of cytosine residues within CpG-rich regions and play central roles in maintaining transcriptional repression and epigenetic stability. Aberrant DNMT activity promotes tumor development through silencing of genes involved in apoptosis, DNA repair, and cell-cycle control [65]. Clinically approved DNMT inhibitors, including azacitidine and decitabine, reverse pathological methylation patterns and restore expression of silenced tumor suppressor genes, thereby improving therapeutic responses in selected malignancies [66]. DNMT inhibition may additionally enhance tumor immunogenicity and increase sensitivity to chemotherapy and immunotherapy. However, direct venom-derived DNMT inhibitors have not yet been convincingly demonstrated or mechanistically validated. At present, evidence linking venom compounds to DNMT modulation remains indirect and insufficient to establish this enzyme class as an active area of venom-derived inhibitor development. Consequently, DNMTs are included here primarily to identify an important translational gap and to highlight future opportunities for investigating whether venom-derived molecules can be engineered or optimized to achieve selective epigenetic regulation in cancer.

#### 3.1.6. Emerging and Underexplored Enzyme Targets: Nucleotide Biosynthesis Pathways

Rapidly proliferating cancer cells depend heavily on nucleotide biosynthesis to sustain DNA replication and repair, making enzymes involved in this network attractive therapeutic targets in oncology. Among these, ribonucleotide reductase (RR) and thymidylate synthase (TS) have achieved substantial clinical relevance through conventional anticancer drugs. RR catalyses the conversion of ribonucleotides into deoxyribonucleotides required for DNA synthesis and repair, and elevated expression of RR subunits has been associated with enhanced proliferation, metastatic behavior, and treatment resistance across multiple tumor types [67]. Likewise, TS catalyses formation of thymidylate (dTMP), a critical precursor for DNA replication, and remains an established target of fluoropyrimidine-based therapies [68,69].

Despite their established importance in cancer pharmacology, there is currently no convincing evidence supporting direct and selective inhibition of RR or TS by venom-derived compounds. Most venom-derived anticancer agents reported to date influence nucleotide metabolism only indirectly through oxidative stress induction, disruption of proliferative signaling pathways, mitochondrial dysfunction, or activation of apoptosis rather than through direct engagement of nucleotide biosynthesis enzymes.

Accordingly, RR and TS are not discussed here as established targets of venom-derived enzyme inhibitors but rather as underexplored opportunities for future investigation. Advances in venom peptide engineering, molecular screening, and structure-guided optimization may enable the identification of venom-inspired scaffolds capable of selectively modulating nucleotide biosynthesis pathways in cancer. The major enzyme classes implicated in cancer progression and representative venom-derived inhibitory compounds are summarized in Table 2.

### 3.2. Mechanistic Basis of Enzyme Inhibition in Anticancer Therapy

The anticancer efficacy of enzyme inhibitors depends on their ability to selectively interfere with enzymatic processes essential for tumor growth and survival. Different inhibitory mechanisms confer distinct pharmacological properties, therapeutic advantages, and resistance profiles.

**(1)**  Reversible Inhibition

Reversible inhibitors interact non-covalently with enzymes and may function through competitive, uncompetitive, or mixed mechanisms. Competitive inhibitors bind directly to catalytic sites and compete with endogenous substrates, as exemplified by many ATP-competitive kinase inhibitors and TS inhibitors [70]. Allosteric reversible inhibitors bind distal regulatory sites, inducing conformational changes that suppress catalytic activity while potentially improving selectivity [71].

**(2)**  Irreversible Inhibition

Irreversible inhibitors form covalent interactions with catalytic residues, resulting in sustained enzyme inactivation. Covalent kinase inhibitors such as osimertinib and ibrutinib exemplifies this strategy [72]. Although irreversible inhibition may enhance potency and duration of action, it also increases the risk of off-target toxicity.

**(3)**  Topoisomerase Poisoning

Topoisomerase poisons represent a distinct inhibitory mechanism in which inhibitors stabilize transient enzyme–DNA cleavage complexes rather than simply suppressing catalytic activity. Persistent DNA strand breaks generated by stabilized cleavage complexes trigger replication fork collapse, genomic instability, and apoptosis [73].

**(4)**  Allosteric Modulation

Allosteric inhibitors suppress enzyme activity by binding sites outside the catalytic pocket, thereby inducing conformational changes that reduce substrate turnover. This strategy may enhance target selectivity and reduce susceptibility to resistance mutations affecting active sites [74].

**(5)**  Prodrug-Based Strategies

Enzyme-activated prodrugs exploit elevated enzymatic activity within the tumor microenvironment to achieve selective drug release. Protease-activated prodrugs cleaved by MMPs, cathepsins, or serine proteases are particularly promising for minimizing systemic toxicity while enhancing intertumoral drug accumulation [75].

**(6)**  Targeted Protein Degradation

Proteolysis-targeting chimeras (PROTACs) represent an emerging strategy in which target proteins are recruited to E3 ubiquitin ligases for proteasomal degradation. Unlike conventional inhibitors, PROTACs eliminate the target protein entirely and may overcome resistance associated with target overexpression or mutation [76].

Despite the success of enzyme-targeted therapies, resistance remains a major clinical challenge. Common resistance mechanisms include target mutation, target overexpression, compensatory signaling activation, enhanced DNA repair, altered drug metabolism, and increased drug efflux mediated by ATP-binding cassette transporters [77]. Current strategies to overcome resistance include rational inhibitor redesign, combination therapies, advanced delivery systems, and targeted protein degradation technologies.

### 3.3. Venom-Derived Mechanisms of Enzyme Modulation

Venom-derived molecules exhibit exceptional mechanistic diversity and often interact with cancer-associated enzymes through unconventional inhibitory pathways.

**(1)**  L-Amino Acid Oxidases (LAAOs)

Snake venom L-amino acid oxidases catalyse oxidative deamination of amino acids, generating hydrogen peroxide and reactive oxygen species (ROS) [30]. Elevated ROS production induces oxidative stress, mitochondrial dysfunction, DNA fragmentation, and apoptosis in cancer cells. Because malignant cells often exhibit impaired redox homeostasis, they may be particularly susceptible to LAAO-mediated oxidative damage.

**(2)**  Venom Phospholipases A_2_

Venom-derived PLA_2_s induce membrane destabilization, phospholipid hydrolysis, and generation of bioactive lipid mediators. These events promote apoptosis, autophagy, mitochondrial dysfunction, and necrotic cell death. Cooperative interactions between PLA2s and membrane-active peptides such as melittin further enhance cytotoxic activity [78].

**(3)**  Disintegrins and C-Type Lectin-Like Proteins

Snake venom disintegrins and C-type lectin-like proteins antagonize integrin-mediated adhesion and signaling pathways involved in metastasis and angiogenesis. By disrupting integrin–ECM interactions, these molecules inhibit migration, invasion, endothelial adhesion, and tumour-associated vascularization while promoting anoikis [79]. Examples such as moojecin, vixapatin, and lebecin have demonstrated anti-metastatic and anti-angiogenic activities in preclinical cancer models, underscoring the therapeutic potential of venom-derived integrin antagonists [80,81].

**(4)**  Kunitz-Type Protease Inhibitors

Kunitz-type inhibitors are multifunctional venom peptides that inhibit serine proteases through reversible competitive interactions with catalytic sites. Beyond protease inhibition, several Kunitz-type peptides interfere with angiogenesis, integrin signaling, and tumor cell migration, thereby exerting broad-spectrum anticancer effects [82].

Venom-derived molecules represent a valuable source of structurally unique enzyme modulators with diverse anticancer mechanisms. Advances in venomics, recombinant peptide production, computational modelling, and targeted drug delivery are expected to accelerate the translation of venom-inspired enzyme inhibitors into clinically relevant anticancer therapeutics.

### 3.4. Comparative Evaluation of Cancer-Relevant Enzyme Targets for Venom-Derived Inhibitor Development

Although multiple enzyme classes have been implicated in tumor progression and discussed individually in the preceding sections, their translational suitability as targets for venom-derived inhibitors is unlikely to be equivalent. Prioritization depends not only on biological importance but also on tumor-selective expression, extracellular accessibility, existing preclinical evidence, and feasibility of selective pharmacological modulation. Enzymes associated with extracellular matrix remodeling and tumor–microenvironment communication presently appear to offer the strongest opportunities for venom-derived intervention. Matrix metalloproteinases, phospholipases A_2_ (PLA_2_), and serine protease-associated systems exhibit recurrent overexpression across multiple tumor types and are directly linked to invasion, angiogenesis, metastatic dissemination, and therapeutic resistance. Importantly, these enzyme systems are extracellular or membrane-associated, making them more accessible to peptide-based venom molecules. By contrast, intracellular enzyme classes including topoisomerases, HDACs, DNMTs, ribonucleotide reductase, and thymidylate synthase remain biologically important but currently show comparatively limited evidence supporting direct venom-derived selective inhibition. For these targets, venom-derived compounds have largely demonstrated indirect pathway modulation rather than validated enzyme-selective mechanisms. Current evidence, therefore, suggests that venom-derived anticancer discovery is most mature for proteolytic and phospholipid-regulating pathways and remains exploratory for epigenetic and nucleotide biosynthesis targets. This distinction supports the prioritization of enzyme systems where biological relevance, target accessibility, and venom-derived mechanistic evidence most strongly converge.

## 4. Structure–Activity Relationships (SAR) and Mechanism of Action of Venom-Derived Enzyme Inhibitors

### 4.1. Snake Venom Protease Inhibitors

Protease inhibitors are an important class of venom molecules that regulate enzymatic reactions involved in fibrinolysis, coagulation, and cellular signaling. In 2017, Thakur et al. reported a detailed analysis of snake venom protease inhibitors, highlighting their structural diversity, biological targets, and therapeutic potential [83]. They deliberated on several structural classes of inhibitors, *viz*., Kunitz-type serine protease inhibitors, cystatin-like inhibitors, and snake C-type lectin proteins (snaclecs). Among them, Kunitz-type inhibitors are among the most extensively studied groups. These inhibitors were biologically selective and comparatively non-toxic as compared to other cytotoxic venom enzymes, thereby making them attractive candidates for therapeutic applications. These small, disulfide-rich peptides inhibit serine proteases involved in coagulation pathways. Interestingly, Mukherjee and co-workers also identified snake venom-derived inhibitors and anti-coagulants, viz., PLA_2_ Isoenzymes and Rusvikunin which were also found to target thrombin, plasmin, factor Xa, and similar related enzymes [84]. Plasmin inhibitors and thrombin-binding peptides, regulate fibrinolytic balance and platelet function, demonstrating precise biochemical specificity. The biological activity of snake venom protease inhibitors is strongly influenced by their structural configurations. Kunitz-type inhibitors display a compact fold stabilized by disulfide bridges, which maintain conformational rigidity essential for enzyme recognition. The reactive-site loop acts as the principal functional domain, inserting into the catalytic pocket of target proteases and mimicking natural substrates. However, structural stabilization prevents irreversible cleavage, allowing sustained inhibition.

The development of venom-derived enzyme inhibitors has increasingly demonstrated that biological activity is governed not only by conservation of the overall protein scaffold but also by precise molecular determinants that regulate target recognition, binding affinity and inhibitory kinetics. Consequently, meaningful SAR analyses require direct correlation between structural features and experimentally measured biological parameters rather than qualitative sequence comparisons alone. Recent biochemical, kinetic and structural investigations of Kunitz-type inhibitors and snake C-type lectins have begun to establish these quantitative relationships, providing valuable guidance for rational optimization of venom-derived therapeutics.

The Kunitz family represents one of the best-characterized examples of SAR among venom-derived protease inhibitors. Although members of this family share a highly conserved disulfide-rich Kunitz fold and exhibit 60–70% sequence identity, they display markedly different inhibitory profiles, indicating that biological activity is determined primarily by localized structural features rather than the conserved scaffold. Comparative biochemical studies have identified the reactive inhibitory loop, particularly the P1 residue and its neighboring amino acids, as the principal determinants of target recognition. These residues directly interact with the catalytic pocket of serine proteases, and even single amino acid substitutions within the reactive loop can produce substantial changes in inhibitory potency while preserving the overall tertiary structure. Rusvikunin (6.9 kDa), Kunitz-type inhibitor isolated from *Daboia russelii*, exemplifies this quantitative SAR. The presence of a conserved Arg residue at the P1 position confers potent inhibition of trypsin (IC_50_ ≈ 50 nM), whereas inhibition of plasmin is approximately 22-fold weaker (IC_50_ ≈ 1.1 μM). Rusvikunin also suppresses thrombin-mediated fibrinogen clotting (IC_50_ ≈ 1.3 μM) but exhibits little or no activity against factor Xa, tissue plasminogen activator or chymotrypsin. These quantitative differences demonstrate that the conserved Kunitz scaffold primarily provides structural stability, whereas the composition and spatial arrangement of residues surrounding the reactive loop determine enzyme selectivity through modulation of steric complementarity, hydrogen bonding and electrostatic interactions within the catalytic site [85,86]. Beyond primary sequence determinants, quaternary organization represents an additional level of SAR regulation. Structural and biophysical analyses demonstrated that Rusviknin forms a stable non-covalent 1:2 complex with Rusvikunin-II while retaining its native secondary structure. Rather than inducing conformational changes, complex formation increases the hydrodynamic size of the inhibitor and significantly enhances anticoagulant activity relative to the individual proteins, indicating that oligomerization improves functional avidity through multivalent target engagement rather than alteration of the inhibitory scaffold [85,87].

Further mechanistic studies revealed that the Rusvikunin–Rusvikunin-II complex contains low-abundance serine protease and phospholipase A_2_ molecules that remain tightly associated with the inhibitor assembly. Although the isolated proteins lack detectable fibrinogenolytic or phospholipase activity, the intact complex exhibits measurable catalytic activity, illustrating that intermolecular assembly generates emergent biological functions not observed in the individual components. Spectrofluorometric titration, equilibrium gel filtration, circular dichroism and dynamic light scattering collectively confirmed stable complex formation with nanomolar binding affinity while preserving the characteristic Kunitz fold. These findings establish protein–protein interactions and multivalent molecular organization as important SAR determinants, demonstrating that pharmacological potency depends not only on reactive-loop composition but also on higher-order structural organization that optimizes target recognition and biological efficacy.

A contrasting SAR is observed in the C-type lectin protein RVsnaclec, which differs fundamentally from Kunitz inhibitors in both structural architecture and inhibitory mechanism. Rather than employing a canonical reactive inhibitory loop for active-site blockade, RVsnaclec selectively targets coagulation factor Xa through reversible uncompetitive inhibition with a Ki of approximately 0.52 μM, indicating preferential binding to the enzyme-substrate complex. Consistent with this allosteric mechanism, RVsnaclec exhibits potent anticoagulant activity while showing no detectable inhibition of trypsin, plasmin, thrombin or chymotrypsin, demonstrating that the C-type lectin scaffold confers remarkable target specificity toward factor Xa. Structural analyses further reveal that RVsnaclec exists as multimeric (αβ)2 and (αβ)4 assemblies containing an unusually small β-subunit (~9 kDa), distinguishing it from previously characterized snake C-type lectins. This unique quaternary organization appears to facilitate selective factor Xa recognition while maintaining negligible cytotoxicity and high protease specificity, highlighting oligomeric assembly as an important determinant of biological activity. In addition, factor Xa inhibition is strongly influenced by the ionic environment, with maximal inhibitory activity observed at 0.25 mM Ca^2+^ and progressive loss of activity at higher calcium concentrations. These findings demonstrate that electrostatic interactions and calcium-dependent conformational stabilization of the enzyme–lectin interface are critical modulators of binding affinity without altering the underlying allosteric mechanism. These observations illustrate that, unlike Kunitz inhibitors where reactive-loop composition primarily governs activity, the pharmacological profile of RVsnaclec is dictated by the combined effects of scaffold architecture, quaternary organization and electrostatic interactions, thereby providing a distinct quantitative SAR model for venom-derived anticoagulant proteins [88].

Structural investigations have been instrumental in elucidating the molecular mechanisms underlying protease inhibition by Kunitz-type inhibitors. High-resolution crystallographic studies of inhibitor–protease complexes have established that members of the Kunitz family employ a conserved canonical binding loop that interacts with the catalytic cleft of serine proteases in a substrate-like manner while resisting proteolytic cleavage. A landmark structural study by [89] determined the crystal structure of the marine-derived Kunitz inhibitor ShPI-1 from *Stichodactyla helianthus* in complex with bovine trypsin at 1.7 Å resolution. This study demonstrated that, despite its evolutionary divergence from mammalian inhibitors, ShPI-1 retains the characteristic BPTI/Kunitz fold and recognizes trypsin through a highly conserved interaction interface. The inhibitor adopts the classical canonical binding mode in which the reactive loop inserts into the catalytic cleft of trypsin, confirming that the overall mechanism of serine protease inhibition is conserved across vertebrate and invertebrate Kunitz inhibitors [89].

Detailed structural comparisons with bovine pancreatic trypsin inhibitor (BPTI) and the amyloid precursor protein inhibitor (APPI) further revealed that residues spanning the P3-P3′ region form the principal interaction surface with trypsin. The reactive P1 lysine residue occupies the S1 specificity pocket of trypsin, where it forms a direct hydrogen bond with Asp189, thereby providing the major energetic contribution to complex formation. This observation corroborates previous crystallographic studies demonstrating that the identity of the P1 residue largely determines inhibitor specificity toward trypsin-like serine proteases. An important contribution of the ShPI-1 structure is the identification of an additional stabilizing interaction mediated by Arg11 at the P3 position. Unlike the proline residue found at the equivalent position in classical mammalian BPTI, Arg11 penetrates deeply into the S3 pocket of trypsin and establishes multiple direct and water-mediated hydrogen bonds together with favorable hydrophobic contacts. Computational free-energy calculations predicted that the replacement of Arg11 with alanine would reduce binding affinity by approximately one order of magnitude, highlighting the significant energetic contribution of this residue to inhibitor binding. The crystal structure also emphasized that inhibitor recognition is not governed solely by the reactive P1 residue. Variations within the prime-side residues (P1′–P4′) and the secondary binding loop modulate the overall interaction network by altering hydrogen bonding, hydrophobic contacts, and water-mediated interactions. Compared with BPTI, ShPI-1 exhibits fewer intermolecular contacts on the prime side and within the secondary binding loop, explaining its relatively weaker affinity despite conservation of the canonical inhibitory mechanism. These findings demonstrate that residues outside the reactive loop substantially influence inhibitor potency and specificity by fine-tuning the stability of the enzyme–inhibitor interface [89].

Snake venom protease inhibitors primarily target enzymes associated with hemostasis and extracellular proteolysis. Major molecular targets include thrombin, plasmin, kallikrein, factor IX and factor X, trypsin-like serine proteases. These enzymes regulate clot formation, fibrin degradation, and platelet activation [90,91]. By binding either to enzyme active sites or precursor zymogens, venom inhibitors interfere with proteolytic cascades essential for physiological regulation. Interestingly, many of these proteases are also implicated in pathological conditions beyond envenomation. Protease-driven remodeling of extracellular matrices and activation of signaling molecules are critical processes in tumor invasion and metastasis, linking venom inhibitor targets to cancer biology. Classical inhibitors function through competitive binding while occupying the catalytic groove and blocking substrate access. Meanwhile, other inhibitors operate through non-enzymatic mechanisms by binding to the coagulation factors or zymogens, thereby preventing activation of downstream proteolytic cascades. Disulfide-stabilized scaffolds ensure that inhibitors remain intact after enzyme interaction, allowing for reversible yet persistent inhibition. This diversity of mechanisms highlights the evolutionary optimization of venom peptides for precise biochemical control.

Although this review focuses primarily on pathophysiology and hemostatic regulation, the described mechanisms have direct implications for cancer treatment. Tumor progression depends heavily on proteolytic enzymes that degrade extracellular matrices, facilitate cell migration, and promote angiogenesis. Proteases such as plasmin and thrombin contribute to tumor invasion and metastatic spread by activating signaling pathways and remodeling tissue environments. Among snake venom-derived Kunitz-type inhibitors, the protease inhibitor PIVL, isolated from *Macrovipera lebetina* trans-mediterranea venom, has demonstrated promising anti-glioblastoma activity. Using the human glioblastoma U87 cell line, [92] showed that PIVL inhibited cell adhesion, migration, and invasion in a concentration-dependent manner without exhibiting cytotoxicity. Functional inhibition of cell migration was detectable at approximately 100 nM, with maximal inhibition observed at 1 μM. Time-lapse video microscopy further revealed that treatment with PIVL reduced the average migration velocity of U87 cells by approximately 32%, from 0.98 ± 0.14 μm/min in untreated cells to 0.67 ± 0.10 μm/min following treatment. Moreover, PIVL inhibited U87 cell migration and invasion with IC_50_ values of approximately 250 nM and 300 nM, respectively. Mechanistically, these effects were attributed to the inhibition of αvβ3 integrin, with weaker effects on αvβ6, αvβ5, α1β1, and α5β1 integrins, and the Arg-Gly-Asn (RGN; residues 41–43) motif of PIVL was identified as a critical determinant of its anti-migratory activity. These findings demonstrate that, beyond protease inhibition, certain Kunitz-type inhibitors can suppress glioblastoma cell motility by disrupting integrin-mediated cell adhesion and migration pathways, highlighting their potential as leads for anti-invasive cancer therapeutics. Snake venom protease inhibitors, by selectively targeting these enzymes, represent promising scaffolds for anticancer drug development. Their structural stability, high specificity, and ability to regulate protease cascades suggest potential use as anti-metastatic agents [92].

### 4.2. Bumblebee Venom-Derived Protease Inhibitor

Qui and co-workers studied a serine protease inhibitor isolated from the venom of the bumblebee *Bombus terrestris* and reported important molecular insights into the molecular cloning and antifibrinolytic activity [93]. Although the primary investigation focuses on antifibrinolytic activity, the structural and mechanistic findings establish a framework that can be extended to cancer-associated protease regulation. The inhibitor identified in bumblebee venom belongs to the Kunitz-type family of serine protease inhibitors, characterized by a compact polypeptide structure stabilized by conserved disulfide bonds. These disulfide linkages are essential for maintaining the inhibitor’s tertiary structure and preserving biological activity under physiological conditions [94]. The study highlights that inhibitory potency is largely determined by the reactive-site loop, a flexible yet structurally constrained region that interacts directly with the catalytic pocket of target proteases. Within this reactive loop, specific amino acid residues surrounding the scissile bond dictate enzyme recognition and binding affinity. The inhibitor mimics a natural substrate; however, instead of undergoing efficient cleavage, its stabilized conformation prevents catalytic turnover, resulting in sustained enzyme inhibition. Structural rigidity provided by cysteine residues and intramolecular bonding ensures that the inhibitor maintains its functional conformation after binding. Minor variations in loop residues can significantly alter specificity, demonstrating that enzyme selectivity arises from precise steric and electrostatic complementarity between inhibitor and enzyme active site. This study therefore established that activity depends on three major structural determinants: (i) conserved cysteine residues forming stabilizing disulfide bridges, (ii) amino acid composition of the reactive site loop, and (iii) conformational stability that balances flexibility for binding with resistance to proteolytic degradation [95]. These features collectively define the structure–activity relationship governing inhibitory efficiency. Biochemical assays identified plasmin as the primary molecular target of the bumblebee venom inhibitor. Plasmin is a serine protease responsible for fibrinolysis through degradation of fibrin clots. The inhibitor demonstrates selective suppression of plasmin activity while showing minimal or no inhibition toward other coagulation-related proteases, indicating a high degree of target specificity [93]. From a biomedical perspective, plasmin represents an important enzyme beyond hemostasis. In pathological conditions such as cancer, plasmin participates in extracellular matrix degradation and activation of additional proteolytic enzymes, including matrix metalloproteinases. These processes facilitate tumor cell migration, invasion, and metastatic dissemination. Consequently, selective inhibition of plasmin has emerged as a promising strategy to interfere with protease-driven tumor progression [96].

Mechanistically, the bumblebee venom-derived inhibitor functions through a competitive inhibition model. The reactive-site loop inserts into the catalytic groove of plasmin, forming a stable enzyme–inhibitor complex that blocks substrate access. Unlike typical substrates, the inhibitor resists irreversible cleavage due to structural stabilization, allowing reversible yet persistent inhibition. Hydrogen bonding interactions and shape complementarity further strengthen binding, preventing catalytic activation. The antifibrinolytic effect observed experimentally results from suppression of plasmin-mediated fibrin degradation. Translating this mechanism to cancer biology, similar inhibition could limit proteolytic remodeling of the tumor microenvironment. By restricting extracellular matrix breakdown and downstream protease activation, such inhibitors may reduce tumor invasion and angiogenesis [97]. Thus, the mechanistic principles identified in the study provide insight into how venom-derived peptides can modulate enzymatic networks involved in disease progression. Although the original research does not directly evaluate anticancer activity, the molecular characteristics described strongly support therapeutic relevance. Cancer progression relies heavily on protease-mediated pathways, and inhibitors capable of selectively targeting these enzymes offer a strategy distinct from cytotoxic therapies [98]. The structural robustness, specificity, and stability of Kunitz-type venom inhibitors make them attractive templates for drug development. Engineering modifications guided by SAR principles could enhance selectivity toward cancer-associated proteases while maintaining resistance to degradation.

### 4.3. Serine Protease Inhibitor

**Figure 3:**
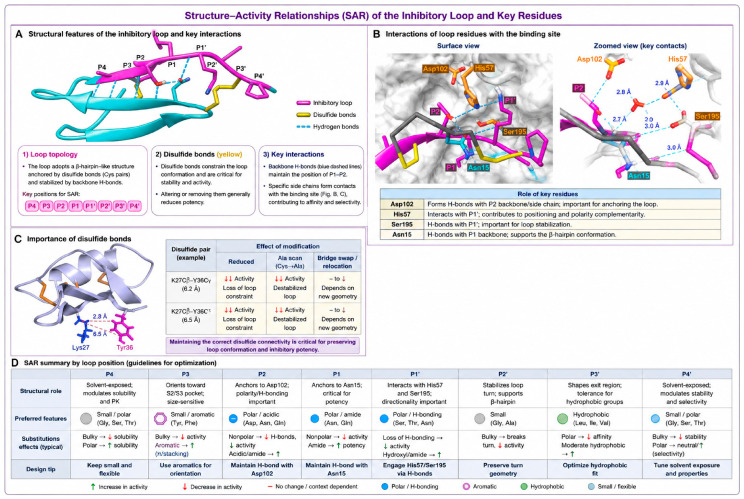
(**A**) AlphaFold modeled structure of SGPI-2 (PDB ID: 2KGH) and (**B**) structural details representing the central part of the enzyme–inhibitor complex. (**C**) Structure of charybdotoxin (PDB ID: 2CRD) showing the Lys-Tyr dyad. (**D**) SAR summary with respect to the loop position.

The research article by Koller et al. explores the molecular features that determine the effectiveness of a canonical serine protease inhibitor, SGPI-2 (Snake Gland Proteinase Inhibitor-2), in regulating enzymatic activity [99]. The study focuses on understanding how structural modifications influence inhibitor stability, enzyme recognition, and resistance to proteolytic cleavage. By applying directed evolution and biochemical characterization, the authors identify key structural elements responsible for maintaining inhibitory function. The activity of the inhibitor is largely governed by its reactive site loop, a specialized structural region that interacts directly with the catalytic pocket of the target protease. This loop behaves similarly to a natural substrate; however, unlike substrates that undergo permanent cleavage, an effective inhibitor preserves its structural integrity after binding. The study demonstrates that amino acid residues positioned around the cleavage site critically influence inhibitory efficiency. Substitutions at these positions alter binding strength, susceptibility to enzymatic processing, and overall inhibitor durability. A major finding of the work is that successful inhibition requires an optimal balance between flexibility and conformational stability. Increased flexibility within the reactive loop makes the inhibitor more prone to enzymatic cleavage, whereas enhanced structural rigidity improves resistance to degradation while preserving strong enzyme binding. Specific side chain interactions and hydrogen bonding networks stabilize the inhibitor–enzyme complex, thereby prolonging inhibitory action. These observations highlight that structural complementarity between inhibitor and enzyme active site is essential for sustained inhibition. Although the study primarily addresses fundamental protein–enzyme interactions, the identified structure-activity relationships have broader biomedical implications. AlphaFold modeled structure of SGPI-2 (PDB ID: 2KGH) is shown in Figure 3. The inhibitory loop is colored in pink, the loop-stabilizing disulfides are shown in yellow, while the hydrogen bonds responsible for loop-stabilizing are indicated as light blue dotted lines. In Figure 3B, the inhibitory loop (pink) is in canonical conformation and occupies the substrate-binding groove of chymotrypsin C, a digestive enzyme belonging to the serine protea ziconotidese family. The catalytic triad is depicted in orange and for better representation, only the inhibitory loop and the adjacent Cys14-Cys17 segment of SGPI-2 (shown in grey) are included, with Asn15 forming several hydrogen bonds that stabilize the loop, illustrated in cyan. Proteolytic enzymes play important roles in cancer progression by facilitating extracellular matrix remodeling, tumor invasion, angiogenesis, and metastasis. Designing inhibitors that maintain high affinity while resisting proteolytic breakdown is therefore a central objective in anticancer drug development. The mechanistic insights provided by this study offer guiding principles for engineering robust protease inhibitors that could be adapted to target cancer-associated proteases.

Venom-derived enzyme inhibitors, particularly Kunitz-type serine protease inhibitors, exhibit conserved structural features that underpin their biological activity [100]. These peptides typically comprise 50–60 amino acids stabilized by three disulfide bonds, forming a rigid structure essential for stability and high-affinity binding. The stability conferred by this disulfide-rich framework allows these peptides to remain active under extreme physiological conditions, including variations in pH and temperature. Their inhibitory mechanism follows a canonical substrate-mimicking model, where the reactive site loop inserts into the protease active site (S3-S3′), forming a stable, reversible enzyme–inhibitor complex that resists complete hydrolysis [101]. The P1 residue is a key determinant of specificity, with lysine or arginine favoring trypsin-like proteases and hydrophobic residues favoring chymotrypsin-like enzymes, while the surrounding P3-P3′ region enhances binding affinity through complementary interactions [102,103]. A key feature of this mechanism is the formation of a highly stable enzyme–inhibitor complex, often with near-stoichiometric (1:1) binding. The strong interaction is maintained by multiple non-covalent forces, including hydrogen bonding, electrostatic interactions, and van der Waals forces. The effectiveness of inhibition is largely governed by the complementarity between the inhibitor’s reactive loop and the enzyme’s active site geometry. Even minor alterations in amino acid residues within this loop can significantly impact binding affinity and inhibitory potency. Despite sequence variability, these functional residues remain conserved, reflecting evolutionary pressure to maintain activity. Structural variations, particularly in cysteine arrangement, contribute to functional diversity, enabling inhibition of multiple protease classes. Additionally, some venom inhibitors exhibit dual functionality by also targeting ion channels, such as voltage-gated potassium channels, thereby enhancing their biological effects.

### 4.4. Charybdotoxin

The reason behind the structural stability of charybdotoxin is a conserved network of disulfide bonds that maintains its compact conformation under physiological conditions. In comparison to classical Kunitz-type protease inhibitors, charybdotoxin lacks a functional protease inhibitory loop and is instead specialized for ion channel targeting. Its biological activity is primarily determined by a surface-exposed binding interface, where key residues particularly, a critical lysine, interact directly with the pore region of potassium channels [104,105].

In potassium channel-blocking peptides such as charybdotoxin-like molecules, SAR is governed by a functional dyad, typically consisting of a lysine residue and a hydrophobic/aromatic residue. Venom-derived peptide inhibitors of voltage-gated potassium (Kv) channels represent one of the best-characterized classes of ion channel modulators and provide valuable insights into quantitative SAR. One of the most significant SAR determinants is the conserved functional dyad, consisting of a positively charged lysine residue paired with a neighboring aromatic residue, typically tyrosine or phenylalanine. This dyad is critical for interaction with the channel pore.

Structural and electrophysiological studies demonstrate that the lysine residue projects into the extracellular pore of Kv channels, where it occludes ion conduction by interacting directly with residues within the selectivity filter, while the aromatic residue stabilizes the toxin–channel complex through hydrophobic and van der Waals interactions. Site-directed mutagenesis consistently shows that substitution of either residue produces a marked reduction in channel affinity, confirming that the functional dyad constitutes the principal pharmacophore responsible for high-affinity Kv channel inhibition. Comparative analyses of scorpion, sea anemone and snake venom peptides further demonstrate that subtype selectivity is primarily determined by amino acid residues surrounding the functional dyad. Although many Kv channel toxins retain the conserved cysteine framework and exhibit similar tertiary structures, relatively small substitutions within solvent-exposed loops significantly alter electrostatic complementarity with the extracellular vestibule of different Kv channel isoforms. These localized structural variations produce measurable differences in affinity for Kv1.1, Kv1.2, Kv1.3 and related channels, illustrating that minor modifications in surface-exposed residues translate directly into altered pharmacological profiles. Zhu and co-workers further highlight that peptide engineering has provided compelling quantitative evidence supporting these SAR principles. Rational modification of surface residues, including residue substitutions, terminal modifications and optimization of electrostatic charge distribution, has generated analogues with substantially improved Kv1.3 selectivity while maintaining high inhibitory potency. These findings demonstrate that relatively small structural changes can markedly reduce off-target inhibition of closely related Kv channel subtypes without disrupting the conserved disulfide-rich scaffold, thereby improving therapeutic specificity. An additional SAR principle emerging from comparative structural analyses is that diverse venom peptide scaffolds including cysteine-stabilized α/β (CSαβ) peptides, Kunitz-type inhibitors and sea anemone toxins can converge on a common mechanism of Kv channel inhibition despite substantial differences in primary sequence and backbone topology. The preservation of the spatial orientation of key pharmacophoric residues enables structurally unrelated peptides to recognize overlapping binding sites on Kv channels with comparable potency. This structural convergence demonstrates that biological activity depends principally on the geometry of the binding interface rather than the overall peptide scaffold [106].

The conserved Lys–Tyr/Phe functional dyad has traditionally been regarded as the principal pharmacophore responsible for potassium channel blockade by α-KTx scorpion toxins. Structural studies of charybdotoxin established this classical SAR model, demonstrating that the Cβ atom of the pore-inserting lysine is positioned approximately 6.2 Å and 6.5 Å from the Cγ and Cζ atoms, respectively, of the neighboring tyrosine residue (Figure 3C). This highly conserved spatial arrangement enables a two-step binding mechanism in which electrostatic attraction between positively charged residues on the toxin and negatively charged regions surrounding the extracellular vestibule promotes initial docking, followed by insertion of the lysine side chain into the selectivity filter. Hydrophobic contacts and hydrogen-bonding interactions involving the adjacent aromatic residue subsequently stabilize the toxin–channel complex, resulting in high-affinity inhibition of potassium ion conductance. Stehling et al. substantially broadened this classical SAR model through the structural and functional characterization of the Amazonian scorpion toxin Tc32. In contrast to canonical α-KTx toxins, Tc32 lacks the conserved pore-blocking lysine that forms part of the functional dyad, yet recombinant Tc32 retained significant Kv1.3 channel blocking activity in electrophysiological assays. This finding demonstrates that the functional dyad, although highly effective, is not an absolute structural requirement for potassium channel inhibition [107]. High-resolution NMR spectroscopy further revealed that Tc32 preserves the characteristic cysteine-stabilized α/β (CSαβ) scaffold, indicating that the conserved disulfide-rich framework primarily functions to maintain the three-dimensional presentation of pharmacologically important surface residues rather than directly mediating channel blockade. Molecular docking studies showed that Tc32 adopts a distinct orientation on the extracellular surface of Kv1.3, where a broader network of positively charged and polar residues distributed across the β-sheet and neighboring loop regions establishes complementary electrostatic interactions with the channel vestibule. Consequently, channel recognition is achieved through a distributed interaction interface rather than insertion of a single pore-occluding lysine residue, illustrating that multiple intermolecular contacts can compensate for the absence of the canonical functional dyad.

Comparison of Tc32 with classical dyad-containing toxins therefore refines the current understanding of SAR within the α-KTx family. While both classes retain the conserved CSαβ scaffold, differences in the composition and spatial organization of solvent-exposed residues generate distinct binding geometries that influence channel affinity and subtype selectivity. These observations indicate that the conserved scaffold serves primarily as a structural platform, whereas the three-dimensional arrangement of charged, hydrophobic and hydrogen-bonding residues determines biological activity. Thus, Kv channel inhibition depends on the overall geometry and physicochemical complementarity of the toxin–channel interface rather than strict conservation of individual amino acid residues.

### 4.5. Chlorotoxin

A key structural determinant in these venom-derived peptides is the surface-exposed functional region which are often termed as the reactive loop or binding interface responsible for mediating direct interaction with the target. In classical protease inhibitors, this region mimics the natural substrate and contains residues that dictate specificity, whereas in toxins such as Chlorotoxin, the functional surface is adapted for binding to membrane-associated proteins including matrix metalloproteinases and chloride channels [108]. The SAR of these peptides is governed by several factors: the disulfide bond arrangement that stabilizes the active conformation, the distribution of surface charges, particularly, positively charged residues that promote interaction with negatively charged cell membranes, hydrophobic patches that enhance binding to membrane targets, and the flexibility and length of loop regions that influence accessibility to binding sites [109]. In chlorotoxin-based systems, even minor modifications in amino acid sequence or conjugation to delivery platforms can significantly improve tumor-targeting ability and binding affinity without disrupting the core disulfide-stabilized scaffold, highlighting the critical role of peripheral residues in target recognition and the overall functional adaptability of these peptides [110]. Furthermore, chlorotoxin exhibits inherent tumor-targeting capability due to its recognition of overexpressed membrane proteins in cancer cells, while sparing normal tissues. Importantly, its core disulfide-stabilized scaffold tolerates chemical modifications such as fluorescent labelling or nanoparticle conjugation without loss of targeting ability, indicating that certain regions remain flexible or non-essential for binding and thus provide valuable opportunities for therapeutic design and targeted drug delivery. Chlorotoxin-derived peptide conjugates, such as tozuleristide (BLZ-100), which are engineered from scorpion venom are utilized for fluorescent labelling of anti-cancer drugs [111]. Tozuleristide is a 36-amino-acid peptide derived from Chlorotoxin, retaining its conserved backbone that enables efficient tissue penetration and high specificity toward tumor-associated targets while maintaining strong binding affinity to enzymes such as matrix metalloproteinases (MMPs). Its structure is stabilized by multiple disulfide bonds that preserve a rigid tertiary conformation essential for receptor binding, thereby enhancing selectivity and resistance to proteolytic degradation in vivo. The peptide displays a positively charged surface that promotes interaction with negatively charged cell membranes and enzyme domains, with specific residues facilitating binding to MMP-2 and interaction with Annexin A2, a membrane-associated protein overexpressed in cancer cells; these interactions rely on precise spatial orientation of functional residues, underscoring the importance of structure–activity relationships. In tozuleristide, conjugation with a fluorescent indocyanine green derivative does not significantly disrupt binding affinity but enhances visualization capability, demonstrating that modifications at non-critical regions can preserve biological activity while adding functionality. Furthermore, its tumor selectivity arises from the ability to recognize overexpressed enzymes and the differential localization of target proteins such as Annexin A2, highlighting that structural features enabling selective interaction with tumor-specific microenvironments are key determinants of its activity [108].

In the case of Chlorotoxin-derived venom peptides, the mechanism of action extends beyond classical enzyme inhibition, involving interactions with cell surface complexes that include MMP-2, chloride channels, and lipid rafts, particularly in glioblastoma cells. These peptides exhibit selective binding to tumor cell surfaces due to their high affinity for overexpressed proteins, enabling targeted recognition. Following binding, the peptide-receptor complex undergoes internalization via endocytosis, facilitating intracellular delivery, especially when conjugated with nanoparticles or vesicles [112]. Additionally, by interacting with MMP-associated complexes, chlorotoxin-derived peptides inhibit extracellular matrix degradation, thereby reducing tumor invasion and metastasis. Their interaction with chloride channels further disrupts ion homeostasis, influencing cell migration and proliferation. When engineered onto extracellular vesicles, these peptides significantly enhance targeted delivery, improving therapeutic efficiency while minimizing off-target effects [113].

### 4.6. Octopus Venom-Derived Enzyme Inhibitors

In structured venom peptides, conserved cysteine residues typically form disulfide bridges that stabilize a rigid scaffold and ensure proper spatial orientation of functional residues; however, in peptides such as Octpep-1, biological activity is driven more by sequence-specific interactions and cellular permeability than by extensive disulfide stabilization, demonstrating that both rigid and flexible frameworks can achieve potent effects depending on functional context [114]. A key determinant of activity lies in surface physicochemical properties, including charge distribution that promotes electrostatic interaction with negatively charged cell membranes, hydrophobic residues that facilitate membrane penetration and intracellular localization, and specific functional motifs that enable interaction with protein targets [115]. Octpep-1 exhibits strong cell-penetrating capability, allowing it to enter melanoma cells and localize near the perinuclear region, highlighting cellular uptake as a critical SAR feature, especially for intracellular and enzymes with significant targets. Additionally, its activity extends beyond single-enzyme inhibition, as it modulates multiple signaling pathways such as the PI3K/AKT/mTOR axis, indicating network-level interactions achieved through subtle amino acid variations. Another important SAR aspect is its synergistic compatibility with other inhibitors, where its structural features allow effective combination with ERK and mTORC1 inhibitors, significantly enhancing anticancer activity and underscoring the importance of complementary binding and pathway targeting in therapeutic optimization [116].

### 4.7. Honeybee Venom Derived Melittin and Phospholipase A_2_ (bvPLA_2_)

**Figure 4:**
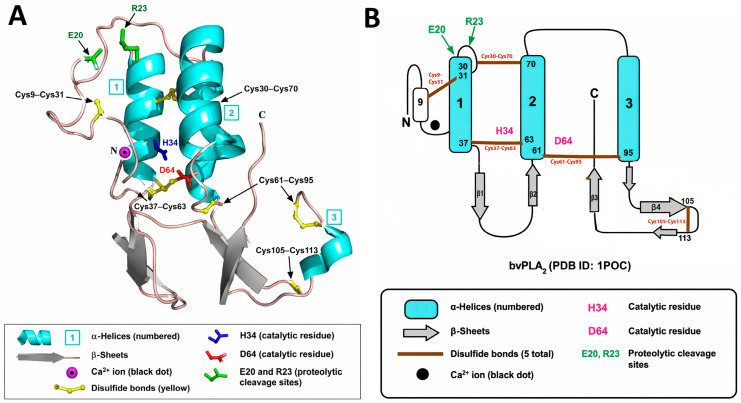
(**A**) Tertiary structures and (**B**) schematic representations of the disulfide bond positions of bvPLA_2_ (PDB ID: 1POC).

Bee venom is a complex biological mixture rich in bioactive peptides and enzymes with significant therapeutic potential, among which melittin (MEL) and bee venom phospholipase A_2_ (bvPLA_2_) are key contributors to anticancer activity [117,118]. Melittin is a 26-amino-acid amphipathic peptide with an α-helical structure characterized by a hydrophobic N-terminal region and a positively charged C-terminal region, enabling strong electrostatic interactions with negatively charged cancer cell membranes and deep insertion into lipid bilayers. This structural arrangement promotes membrane destabilization, pore formation, and efficient cytolysis, while its sequence composition, charge distribution, and optimal length govern its cytotoxicity and selectivity. Unlike other venom derived inhibitors, the lack of cysteine residue in melittin results in the absence of disulfide bonds, which enhances structural flexibility, allowing for adaptation to diverse membrane environments but also contributing to nonspecific toxicity, which can be mitigated through sequence modifications or delivery systems such as nanoparticle conjugation [15]. Melittin can also self-associate into oligomers, further enhancing pore-forming ability and cytotoxic efficiency, and interact with intracellular targets, contributing to broader anticancer mechanisms. In contrast, bvPLA_2_ is a calcium-dependent enzyme that hydrolyses phospholipids at the sn-2 position, with catalytic activity driven by key residues such as histidine and aspartate and maintained by disulfide bond-stabilized structural integrity [119,120]. Its specific amino acid sequence and three-dimensional conformation enable selective interactions with cancer and immune cells, while enzymatic hydrolysis generates bioactive lipid mediators like lysophospholipids and arachidonic acid that influence inflammation and tumor progression in a context-dependent manner [121]. The tertiary structures and schematic representations of the disulfide bond positions of bvPLA_2_ are shown in Figure 4. The disulfide bonds are highlighted in yellow and the purple colored sphere represents the essential Ca^2+^ ions. The catalytically important amino acid residues H34 and D64 in bvPLA_2_ are labelled while the E20 and R23 mark the proteolytic cleavage sites of pWT by thermolysin. The α-helices are represented by the numbered boxes while the β-sheet structures are shown by grey arrows. In the second diagram (Figure 4B), the black dot indicates the position of the Ca^2+^ ions and the positions of the five disulfide bonds are highlighted as brown lines. Amongst the five disulphide bonds three conserved disulfide bonds are shown as C9–C31, C30–C70, and C61–C95.

Melittin exerts its anticancer effects through multiple interconnected mechanisms closely tied to its amphipathic α-helical structure, beginning with membrane insertion into lipid bilayers that leads to pore formation, followed by leakage of intracellular contents, and finally rapid tumor cell lysis [118]. It further promotes apoptosis via mitochondrial pathways by modulating apoptotic regulators, including downregulation of Bcl-2 and activation of Bax and caspases, while simultaneously inhibiting key cancer signaling pathways such as PI3K/Akt, RAS/MAPK, and NF-κB, thereby suppressing proliferation and survival [122,123,124]. Melittin also interferes with cell cycle progression through interactions with cyclins and CDK2, inducing arrest at G0/G1 or G2/M phases, and exhibits anti-angiogenic and anti-metastatic effects by inhibiting neovascularization, tumor invasion, and modulating the tumor microenvironment [117]. In addition, it activates immune-related mechanisms, including NLRP3 inflammasome-mediated pyroptosis, release of pro-inflammatory cytokines, and induction of immunogenic cell death marked by ATP, HMGB1, and calreticulin release, which enhance dendritic cell activation and T-cell-mediated responses, effectively converting immunologically “cold” tumors into “hot” ones. Its ability to regulate gene expression, including interactions with DNA and modulation of miRNA and lncRNA, further contributes to altered tumor behavior [125]. In therapeutic applications, melittin is often incorporated into nanocarrier systems such as liposomes, micelles, and polymeric platforms to reduce hemolytic toxicity, improve pharmacokinetics, and enhance tumor selectivity via passive and active targeting, and is also used in combination therapies to overcome drug resistance and improve efficacy. Complementing these effects, bee venom phospholipase A_2_ (bvPLA_2_) acts through enzymatic hydrolysis of membrane phospholipids, leading to membrane degradation, apoptosis, and necrosis, while also exerting immunomodulatory functions by enhancing antigen presentation through MHC I and II pathways and activating CD4^+^ and CD8^+^ T cells. It further regulates the tumor microenvironment by modulating inflammatory mediators such as TNF-α and IL-6 and disrupting signaling pathways essential for tumor survival, although its lipid products may exhibit context-dependent effects on tumor progression [118]. Notably, melittin and bvPLA_2_ act synergistically, with melittin initiating membrane disruption and bvPLA_2_ amplifying phospholipid degradation, resulting in enhanced cytotoxicity and significantly improved anticancer efficacy compared to their individual actions. The integrated relationship between structural features, mechanisms of action, and cancer applications of the venom-derived enzyme inhibitors discussed in this section is summarized in Table 3.

## 5. Computational Approaches and Mechanistic Insights in Venom-Derived Enzyme Inhibitor Discovery

Computational approaches have become an integral component of modern drug discovery owing to advances in computing power, algorithm development, and the growing availability of biological and chemical datasets. These methods complement conventional experimental screening by improving the efficiency of candidate identification, reducing costs, and facilitating the prioritization of compounds for laboratory validation. Within venom-based drug discovery, computational tools have supported the identification, characterization, and optimization of bioactive molecules with potential anticancer activity. Predicting peptide–protein interactions (PPIs), however, remains challenging because venom-derived molecules exhibit substantial structural diversity and biochemical complexity, including disulfide-rich peptides and enzymatic proteins (see Table 4). Recent developments in machine learning, molecular model-ling, and structural bioinformatics have improved the prediction of structure-function relationships, molecular targets, and binding specificity, thereby supporting the rational discovery and optimization of venom-derived enzyme inhibitors for cancer therapy [126,127]. Table 4 summarizes structurally defined venom-derived molecules that have been isolated, purified, recombinantly produced, or chemically synthesized. Studies based solely on crude venoms or partially purified venom fractions were excluded because they provide substantially lower levels of molecular characterization.

Computational chemistry and bioinformatics can now go through large venom libraries relatively quickly and predict characteristics of the target binding, its structural binding mechanisms, and the pharmacokinetics parameters of the venom in advance of any extensive in vitro testing. The generic approach normally relies on the support of computational approaches, such as the use of models based on machine learning, ligand-based or sequence-based drug design and structure-based drug design approaches [137,138]. Targeting a specific cancer associated biological target, finding optimum interactions between natural ligands (e.g., venom peptides) and the target is typically their first goal in structure-based drug discovery. Molecular docking and molecular dynamics (MD) simulations are essential tools in these approaches for achieving high resolution.

However, when the 3D structural data of the biological target is not available, ligand-based and sequence-based drug discovery approaches such as pharmacophore mapping and Quantitative Structure–Activity Relationship (QSAR) modeling are extremely useful [139]. In order to ascertain the essential molecular characteristics that underlie biological activity, these methods rely on the examination of known active substances. This allows them to forecast and optimize potential venom-derived ligands that are new based on their chemical and sequence relationships to natural compounds that have already been identified. Furthermore, the emergence of complex models that facilitate both ligand/sequence-based and structure-based drug discovery has been made possible by recent developments in machine learning, notably deep learning. The predictive modeling of key pharmacological properties such as binding affinities has also been aided by these computational breakthroughs, which also include the modeling of absorption, distribution, metabolism, excretion and toxicity (ADMET). To overcome the high failure rate of new drugs in early phase development, which historically has been less than 10% of the initial ideas making it to Phase I clinical trials, it is crucial to accurately predict ADMET properties at computational stage [140,141,142]. Combination of several computational techniques is logically filtering and refining venom poisons, bridging the gap between unprocessed natural libraries and promising therapeutic options. Computational studies (see Table 5) show that the peptides found in venom are mostly involved in membrane disruption, enzyme inhibition, or ion channel modulation.

### 5.1. Sequence-Based Approaches and Machine Learning Screening

Sequence-based methods anticipate interactions with target proteins by utilizing the intrinsic physicochemical characteristics, amino acid composition, and evolutionary information of peptides. Early techniques, such CGKronRLS [148] and NRLMF [149], required known interaction scores as input characteristics and mostly depended on similarity matrices. Computing large dimensional similarity matrices was very complex, causing significant processing bottlenecks [150]. In the last decade, the field has quickly progressed to Deep Learning (DL) and their advanced ensemble algorithms, which overcome such limitations by learning more complex sequence information independently. The anticancer peptides (ACPs) can now be directly classified based on their primary sequence, such as DeepACP, mACPpred, and sequence-based Support Vector Machines (SVMs) [151,152]. The complexity of venom proteomes makes these algorithms good primary screening methods. In this regard, ref. [153] developed a hybrid feature model called iACP-GAEnsC, which was used to assess the extracted descriptors based on an ensemble classification approach with genetic algorithm. A target-based approach called TargetACP was developed by [154] that was based on both evolutionary and sequential approaches to extract numerical features from a peptide sample. Both iACP-GAEnsC and TargetACP had high classification rates (accuracy, sensitivity, specificity, and MCC) but suffered from high computational cost and poor practicality. Traditional models face challenges such as the need for manual feature selection and representativeness, and the inability to efficiently scale large data sets, with high training capabilities. To overcome the shortcomings of traditional models, Deep Neural Networks (DNN) provide automatic feature representation without human involvement, high training capabilities, and can scale large data sets efficiently. For instance, ref. [155] proposed a long short-term memory (LSTM) method, which directly learned the ACP discrimination by using k-mer matrices and binary profiles. ACP-MHCNN is an improved CNN model developed by [156] which extracted the features using composite physicochemical, sequential and evolutionary methods. To effectively represent cancerous mutations, ref. [157] applied a skip gram-based distributed approach (Mut2Vec). Ref. [158] proposed a word embedding based hybrid feature vector to predict tumor necrosis factors by the name of TNFPred. To tackle the difficult task of predicting selective anticancer peptides (ACPs) and develop them for targeted drug delivery against cancer. Ref. [159] propose a novel Deep Neural Network (DNN) model cACP-DeepGram that classifies peptide sequences based on FastText based word-embeddings. This optimized model outperforms existing predictors by an approximate 10% and even DNNs which excel with large training samples have demonstrated their ability to yield excellent prediction accuracy over small training sets without overfitting in recent predictors, including Deep-AntiFP, 2L-piRNADNN, iRSpot-SPI, and iPredCNC.

In recent years, several studies have shown that biotoxins from snakes, spiders, bees, scorpions, wasps, and ants exhibit notable anti-tumor properties [160,161,162,163]. Beyond individual peptide reports, ensemble machine learning frameworks have been used to model peptide-cancer protein relationships using sequence-derived and physicochemical descriptors. For example, approaches combining Random Forest, XGBoost, and stacked autoencoder-deep neural networks (SAE-DNN) have been applied to venom-derived peptide datasets from *Calloselasma rhodostoma*, integrating features such as amino acid composition, intrinsic disorder, and physicochemical properties to predict interactions with cancer-associated proteins [164]. In this context, XGBoost achieved the best performance (accuracy: 0.859, precision: 0.663, ROC-AUC: 0.697), while network enrichment analysis identified key oncogenic hub proteins, including ESR1, GOPC, and BRD4, associated with tumor hallmarks and cancer-related pathways [164].

Similarly, Almeida et al. [12] applied multiple AI-based prediction tools to previously reported snake venom anticancer peptides, integrating structure prediction (ColabFold [165], PEP-FOLD [166]), anticancer activity prediction (AntiCP 2.0 [167], MLACP 2.0 [168], AI4ACP [169]), hemolytic profiling (HemoPI 2.0 [170], HLP-pred-Fuse [171]), and toxicity assessment (CSM-Toxin [172], ToxinPred3.0 [173]) with at least two predictors per category for cross-validation. Rather than focusing on methodological detail, the study collectively illustrates the broader limitation that strong in silico predictions across multiple platforms do not consistently translate into experimental confirmation, reinforcing concerns highlighted in recent assessments of computational peptide discovery regarding the need for experimental validation despite high predictive performance [151,174,175]. Consequently, while machine learning based virtual screening coupled with ADMET filtering improves prioritization efficiency in large venom peptide libraries, its applicability remains constrained by dataset imbalance, limited venom-specific training data, and incomplete experimental annotation [176].

### 5.2. Structure-Based Approaches and Molecular Docking

While sequence-based models excel at quick screening, structure-based models such as molecular docking can help to understand the exact mechanism of binding at atomic level [177]. Other than the obvious requirement of high resolution, there is a need for high level tools that can model large conformational changes as peptides are highly flexible molecules, and they are required for structure-based prediction; CABS-dock [178] is a blind peptide-protein docking tool, and HADDOCK (data-driven docking) [144], and Rosetta FlexPepDock [179] are tools required for that purpose. The prediction of peptide-receptor complexes has been further revolutionized by the introduction of deep learning in structural biology through AlphaFold-Multimer, which has further improved the accuracy of predictions in this field [180].

These structural tools have been important for understanding how peptides interact with particular enzymes that drive cancer. In silico evaluations using the CABS-dock platform, for instance, demonstrated binding of peptides from the venom of the *Nemopilema nomurai* jellyfish to enzymes called MMPs that are crucial in the metastasis of tumors [178]. The peptide DN26779_N had an excellent binding energy of −13.3 kcal/mol with MMPs. Structural models also showed that this peptide can effectively inhibit the invasion of cancer cells by establishing numerous hydrogen bond networks (such as at THR154 and GLU201) and van der Waals interactions close to the catalytic zinc ion of the MMPs, thus preventing them from accessing the substrate [178].

In the same study, docking to the enzyme phospholipase A_2_ (PLAs A_2_) which is involved in phospholipid hydrolysis and inflammatory cascades showed strong binding for DN26779_N (−12.6 kcal/mol). The peptide uses hydrophobic interactions to reduce its interaction with water, thus stabilizing the peptide in the active site of the PLA_2_. In silico structural characterization of other venom derivatives with specific affinity to MMP-2, such as chlorotoxin from scorpion venom, has also been carried out and proved to inhibit specifically the MMP-2 and to prevent the degradation of ECM. Likewise, the peptides DN26796_N and DN26796_Q were reported and molecular docking was carried out to predict their binding sites and binding affinities to specific target proteins by using advanced machine learning algorithms [181].

### 5.3. Targeting Cancer-Associated Hub Proteins and Ion Channels

Beyond degrading enzymes, venom peptides have been computationally mapped to specific oncogenic hub proteins and ion channels. Pathway enrichment and network topologies predicting the targets of *Calloselasma rhodostoma* venom peptides successfully identified (BRD4), (ESR1), and Golgi-associated PDZ and coiled-coil motif-containing protein (GOPC) as primary interactive targets [164]. BRD4, an epigenetic reader regulating cancer gene expression, and ESR1, heavily implicated in metastatic potential, represent highly specific targets for these natural peptides.

Furthermore, scorpion venom peptides are well-documented blockers of voltage-gated ion channels (e.g., Na^+^, K^+^, Cl^−^), which are vital for tumor volume regulation and migration. Toxins such as BmKKx2 from *Buthus Karsch* target hERG potassium channels, while chlorotoxin targets chloride channels in glioma cells mechanisms that have been extensively modelled in silico to fully map their anti-proliferative effects [182].

### 5.4. Molecular Dynamics (MD) and ADMET Profiling

The docking poses are validated and the drug-likeness of venom peptides is tested through Molecular Dynamics (MD) simulations and software such as GROMACS, AMBER and Normal Mode Analysis (NMA) via iMODS. MD simulations have played a crucial role in the last five years in determining the thermodynamic stability of peptide-protein complexes, including the disruption of the oncogenic p53-MDM2 interaction by staple peptides to restore apoptotic pathways [183]. Chemical reactivity and stability of soft molecules: In the case of venom compounds, their DFT calculated energy gap between HOMO and LUMO confirmed their chemical reactivity and stability as soft molecule. The structural flexibility and stability of these complexes over time were also confirmed by molecular docking simulations based on Normal Mode Analysis (NMA), which have low eigenvalues representing high motion stiffness and resistance to deformation [178].

In addition, ADMET profiling is essential to predict translational barriers before entering clinical trials. For example, the ADMET screening of the *Nemopilema nomurai* peptides predicted high human intestinal absorption (HIA > 0.1), without the inhibition of cytochrome P450 enzymes (CYP1A2/CYP3A4), and unable to penetrate the blood-brain barrier [178]. This indicates a highly favorable safety and pharmacokinetic profile for peripheral tumor targeting.

Computational tools have significantly advanced venom-derived enzyme inhibitor discovery, but their reliability varies across stages of the workflow and is constrained by peptide structural complexity. Structure prediction methods such as Al-phaFold2 and ColabFold improve modelling of venom peptides; however, performance decreases for disulfide-rich scaffolds, flexible loop regions, and non-canonical folds, where accurate disulfide pairing and local conformational variability remain difficult to resolve, reinforcing the need for experimental validation [184,185]. Docking approaches further provide mechanistic insight but differ in flexibility treatment: rigid and semi-flexible tools such as AutoDock Vina and Glide enable rapid screening yet often miss induced-fit effects, whereas HADDOCK 2.2, CABS-dock, and Rosetta 3 Flex-PepDock incorporate increasing structural flexibility and experimental restraints, improving realism at higher computational cost [186,187].

Molecular dynamics (MD) simulations (e.g., GROMACS, AMBER) provide the most detailed assessment of complex stability through time-dependent conformational sampling and solvent effects, but remain computationally intensive and are therefore limited to secondary validation [188]. In parallel, machine learning models (e.g., DeepACP, MLACP 2.0, AntiCP 2.0, AI4ACP, TargetACP) enable the rapid sequence-level classification of anticancer potential but are restricted by limited venom-specific datasets, class imbalance, and inconsistent annotation, reducing generalizability and increasing overfitting risk [189]. Suffice to say no single method is sufficient; an integrated pipeline combining machine learning for screening, docking for interaction hypotheses, MD for stability assessment, and experimental validation offers the most reliable framework, with each approach contributing complementary strengths in scalability, interpretability, and biophysical accuracy.

### 5.5. Bridging the Gap in Rational Drug Design

**Figure 5:**
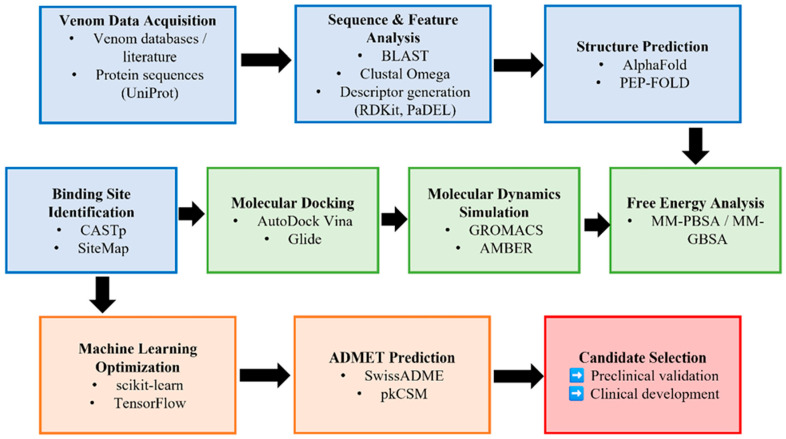
Integrated computational workflow for venom-derived enzyme inhibitor discovery. The 10-stage pipeline is color-coded by methodological phase: structural biology and target preparation (blue, Stages 1–4); molecular simulation and binding dynamics (green, Stages 5–7); machine learning optimization and ADMET profiling (orange, Stages 8–9); and final candidate selection for preclinical/clinical validation (red, Stage 10).

The general paradigm of in silico drug discovery was long dominated by models of small molecule drug-target interactions (DTI), but the current trend is to combine both sequence- and structure-based models of complex biologicals. Nowadays, a rapid sequence-based ML approach is used for screening large natural venom libraries to find potential anticancer peptides and is immediately followed by structure-based docking and MD simulations to validate the molecular mechanism of action. The result is a computational pipeline that is both holistic and is able to connect the dots between high throughput screening and targeted rational drug design as illustrated in Figure 5.

## 6. Preclinical and Clinical Evidence

The transition of venom-derived enzyme inhibitors from computational models to biological systems has led to significant preclinical evidence of their efficacy as anticancer compounds. These compounds offer a comprehensive strategy for cancer therapeutics, inhibiting crucial survival pathways, cellular adhesion, and tumor microenvironments. Despite the wide range of structurally diverse venom-derived bioactives identified as presented in Table 4, only a limited subset has progressed to clinical evaluation (Table 2), highlighting a significant gap between molecular discovery and therapeutic translation.

### 6.1. In Vitro Efficacy: Inhibiting Proliferation and Inducing Apoptosis

In vitro and preliminary in vivo studies have consistently demonstrated the anticancer activity of venom compounds derived from snake, spider and jellyfish, including induction of apoptosis, modulation of oxidative stress, and inhibition of metastatic signaling pathways [134,190,191,192]. L-amino acid oxidases (LAAOs) and PLA_2_s are the key mediators of apoptotic cell death of snake venom. Upon binding to the membrane of cancerous cells, LAAOs induce high concentration of hydrogen peroxide (H_2_O_2_) which disrupt the redox system and trigger intrinsic apoptosis through ROS mediated pathway [193,194] This is characterized by the depolarization of the mitochondrial membrane, release of cytochrome c, and the modulation of the Bcl-2 family (upregulation of pro-apoptotic Bax and downregulation of anti-apoptotic Bcl-2) [195,196].

Scorpion venoms also stops tumor progression by inducing cell cycle arrest. *Androctonus crassicauda* venom triggers arrest in the S phase, while *Buthus martensii* induces G1 and S phase arrest [182], heavily mediated by the upregulation of p53 and p21 inhibitors [197]. The peptide BmKn-2 specifically reduces Bcl-2 and triggers caspases 3 and 9, facilitating mitochondrial-dependent apoptosis in human cells [197]. Moreover, disintegrins non-enzymatic, cysteine-rich peptides derived from snake venom metalloproteinases (SVMPs) reported by [198] are strong inhibitors of integrins (e.g., αvβ3, α5β1). Because integrins are critical for tumor cell attachment to the extracellular matrix (ECM), their blockade by disintegrins such as Acurhagin-C and Lebein induces “anoikis” a form of detachment-induced apoptosis [199,200]. Ref. [201] discovered a new peptide, AATP, from *Haliotis discus hannai*, which has the ability to inhibit matrix metalloproteinases (MMPs). This inhibition was able to block the MAPK and NF-κB signaling pathways, and in the end resulted in less metastasis from the tumor cells. The heterodimeric phospholipase A_2_, crotoxin (CroTx), from the venom of *Crotalus durissus terrificus* showed high and specific cytotoxicity against various human cancer cell lines, including glioma (GAMG, IC_50_ < 0.5 μg mL^−1^; HCB151, IC_50_ = 4.1 μg mL^−1^) and pancreatic (PSN-1, IC_50_ = 0.7 μg mL^−1^; PANC-1, IC_50_ < 0.5 μg mL^−1^) cancer cells, while being not toxic to normal human keratinocytes (HaCaT) and mouse fibroblasts (3T3), demonstrating tumor selectivity [134]. The mechanism by which CroTx caused DNA damage involved H2AX phosphorylation, while CroTx did not have any apparent effect on cell-cycle progression but did induce apoptosis of the cells [134]. Similarly, purified NNLAAO70 from *Naja naja* venom was found to be highly cytotoxic to A549 human lung adenocarcinoma cells (IC_50_ = 0.89 μg mL^−1^), with around 60% cell viability being reduced at 1 μg mL^−1^. Catalase co-treatment also significantly decreased cytotoxicity by almost 50% indicating that hydrogen peroxide-dependent ROS production is a significant mediator of cytotoxicity [133]. However, due to the lack of non-malignant control cells, the tumor selectivity and systemic safety were not determined, restricting its translatability.

Similarly, crude venoms of Egyptian snakes such as *Cerastes cerastes*, *Cerastes vipera*, *Naja haje*, *Echis pyramidum* and *Echis coloratus* showed significant cytotoxicity against MCF-7 breast and HepG2 hepatocellular carcinoma cells with lower IC_50_ values in MCF-7 (3.48–4.49 μg mL^−1^) compared to HepG2 (4.32–217.90 μg mL^−1^), reflecting cell-type dependent susceptibility [202]. Significant changes in oxidative stress and cytotoxicity-associated biomarkers such as LDH, MDA and GSH were also observed in the presence of cytotoxicity [202]. However, the use of “crude” venom mixtures without defining the active bioactive components and the lack of evaluation in normal cell lines prevented any solid conclusions on tumor selectivity or structure–activity relationships from being drawn [202]. The 24-h IC_50_ of *Agkistrodon acutus* venom component I (AAVC-I) against HSC-3 oral squamous cell carcinoma cells was 8.86 μg mL^−1^ in another mechanistic study [203]. AAVC-I suppressed the Keap1/Nrf2 antioxidant signaling pathway, decreased mitochondrial membrane potential, released cytochrome c, activated caspase-9 and caspase-3 and downregulated Bcl-2, which led to ROS-mediated mitochondrial apoptosis [203]. However, the lack of non-malignant cell controls and of a detailed in vivo safety evaluation that would allow for the evaluation of its tumor selectivity and translational applicability.

### 6.2. In Vivo Models: Suppressing Metastasis and Angiogenesis

Metastasis and angiogenesis are the two primary causes of cancer death, and pre-clinical in vivo models have proven the venom compounds’ capacity to block these cascades. The disintegrins with the RGD (Arg-Gly-Asp) motif are competitive inhibitors of integrin binding on tumor and endothelial cells [204]. The in vivo murine models used (e.g., B16F10 melanoma) showed that lung tumor colonization was significantly reduced following intravenous injection of disintegrins (see Table 6) such as Flavoridin and r-DisBa-01 [205,206]. This anti-metastatic effect is through the inhibition of binding of tumor cell to fibronectin and vitronectin in the ECM, thus stopping extravasation and invasion.

Likewise, crotamine, a CPP isolated from *Crotalus durissus terrificus*, was found to specifically localize in proliferating tumor cells and to show that growth of these cells and tumor burden was significantly inhibited with improved survival rates, and with no detectable toxicity to normal tissues, upon tumor implantation [207]. Similarly, crude venom from *Nemopilema nomurai* showed anti-metastatic activity by inhibiting the epithelial–mesenchymal transition (EMT) by blocking both smad- and NF-κB-signaling pathways and restoring E-cadherin expression, but the bioactive compound in the venom that was responsible for this activity has not yet been determined [191].

Venom peptides have been used directly as anticancer agents, but they have also proved useful as delivery agents to target tumors. A cell penetrating peptide (CPP) derived from the spider venom Lycosin was used to selectively target functionalized gold nanoparticles and nanorods to tumor cells, increasing the cellular uptake, promoting efficient near-infrared (NIR) mediated tumor ablation, achieving favorable systemic clearance, and demonstrating minimal in vivo toxicity [208].

All these studies clearly demonstrate complementary actions of venom-derived compounds in suppressing the progression of tumors by inhibiting metastasis, angiogenesis, EMT, and improving the delivery of drugs to the tumor (see Table 6). However, these promising preclinical results have not yet overcome a number of challenges for clinical translation. Many venom peptides are poorly bioavailable when given orally, are rapidly degraded by enzymes in plasma, have short t½s and may become immunogenic upon repeated administration [209]. Thus, efforts have been made to enhance their pharmacokinetic profiles by developing new drug delivery systems. The treatment efficiency of recombinant disintegrins has been greatly improved by liposomal encapsulations, giving greater inhibition of tumor growth and increased survival at the same doses as the free peptide [210]. Similarly, peptide engineering methods such as cyclization, PEGylation, and attachment of nanomaterials, including gold nanoparticles, have increased the stability and the duration of systemic exposure, and the tumor-specific accumulation of peptides [208,211]. Additionally, chlorotoxin-based formulations of nanoparticles for glioblastoma imaging and therapy have been successfully developed clinically, further highlighting the clinical translational potential of venom-derived macromolecules for rational delivery [212]. Standardized preclinical models, thorough pharmacokinetic and toxicological characterization, and optimization of delivery systems, therefore, should be a focus of future research to ensure successful clinical translation.

### 6.3. Clinical Progress

Despite having significant preclinical data that makes venom enzymes and peptides promising candidates for the treatment of various cancers, the clinical translation of this research has been stymied by a number of factors such as systemic toxicity, poor pharmacokinetics, immunogenicity, and inadequate tumor-specific delivery. As a result, there are only a few venom molecules that have advanced to the clinical trial stage and many that are still in preclinical development.

As discussed in Section 4.5, tozuleristide (BLZ-100) is the most advanced near-infrared fluorescent peptide derived from the venom of the deathstalker scorpion (*Leiurus quinquestriatus*) and conjugated to a near-infrared fluorophore. It is a matrix metalloproteinase-2 (MMP-2) and chloride channel-associated complexes targeted which is used for intraoperative fluorescence-guided imaging, not as a cytotoxic therapeutic. In each of the multiple Phase I clinical uses of tozuleristide in the treatment of breast cancer, skin malignancies (basal cell and squamous cell carcinoma and melanoma) and pediatric central nervous system (CNS) tumors, tozuleristide was found to be very safe, with no dose limiting toxicities or treatment related serious adverse events [110,111]. An optimized dose of 15 mg/m^2^, in the case of pediatric CNS tumors, resulted in intraoperative fluorescence in 69.6% of cases, and the ex vivo imaging had a sensitivity of 81% and a positive predictive value of 93% for distinguishing tumor from normal tissue [213]. This data has aided its development to a continuing pivotal Phase II/III clinical trial (NCT03579602) for use in neurosurgery with fluorescence guidance [214].

A second clinically tested venom extract is VRCTC-310 (purified crotoxin), a cardio-toxin-based neurotoxin from the venom of the *C. durissus terrificus*. VRCTC-310 was the first venom-derived product to be studied in a Phase I oncology trial, and was found to be well tolerated in patients with advanced refractory malignancies with an MTD of 0.017 mg kg^−1^. Reversible neurological side effects, such as transient diplopia and palpebral ptosis, were seen in patients receiving treatment, along with injection site pain and eosinophilia; some patients stabilized on treatment [215]. However, the subsequent case reports showed good local regression after intratumoral administration in recurrent SCC [216] and locally advanced breast cancer [217], and these findings are still anecdotal and need to be confirmed in a larger controlled study. Despite the potent preclinical antitumor activity of crotoxin in terms of membrane disruption, induction of apoptosis and inhibition of the EGFR-associated signaling, most of its developmental efforts have been limited to the VRCTC-310 formulation. Similarly, the main peptide of the venom of *A. mellifera*, melittin, shows broad-spectrum anticancer activity by permeabilizing the membrane, inducing mitochondrial dysfunction and apoptosis. But only limited clinical translation of its intrinsic hemolytic toxicity. Melittin-based formulations have shown significant improvement in its therapeutic index in pre-clinical studies, but there is no registered clinical trial using melittin-based formulations in oncology in humans.

There is still limited clinical evidence of the use of venom as an anticancer agent. Tozuleristide is the most clinically advanced candidate, but it is used for imaging purposes at this point, while VRCTC-310 is the first enzyme venom therapeutic candidate, but its therapeutic results have been limited. The majority of other venom-derived peptides (melittin, crotamine, disintegrins and L-amino acid oxidases) are still in the preclinical development phase, calling for new peptide engineering, delivery and suitable clinical trials to bring the promising results obtained in the lab to effective cancer therapies.

### 6.4. Chemo-Sensitization and Combination Therapies

An emerging paradigm in venom research is the synergistic application of venom peptides with established chemotherapeutics. Melanoma cells are notorious for acquiring resistance to standard drugs like methotrexate (MTX) and cisplatin. However, preclinical data indicates that Acurhagin-C acts synergistically with MTX to increase anti-proliferative activity in B16F10 and SK-MEL-1 cells, forcing the tumor cells into S-phase arrest and increasing their sensitivity to the chemotherapy [218].

Similarly, the C-type lectin Macrovipecetin, when combined with cisplatin, significantly enhanced apoptosis in SK-MEL-28 melanoma cells [219]. This combination effectively impaired cell adhesion and migration while decreasing AKT phosphorylation and regulating the MAPK (ERK1/2) survival pathways. Because targeted therapies (such as BRAF and MEK inhibitors) frequently encounter acquired resistance due to the high mutational load of melanomas, utilizing venom peptides as sensitizing adjuvants represents a highly rational therapeutic pathway.

### 6.5. Translational Challenges and Future Perspectives

Despite compelling in vitro and in vivo efficacy, the clinical translation of venom-derived peptides faces notable pharmacokinetic hurdles. Venom peptides particularly hydrophilic molecules with multiple disulfide bridges often suffer from low oral bioavailability, rapid proteolytic degradation in the gastrointestinal tract, and short systemic half-lives [209]. Furthermore, their xenogenic nature raises concerns regarding immunogenicity and the stimulation of neutralizing antibodies upon repeated administration.

To overcome these barriers, advanced drug delivery systems are being actively explored. Liposomal encapsulation has proven highly effective; for instance, liposome-packaged vicrostatin (a recombinant disintegrin) significantly delayed tumor growth and increased survival in MDA-MB-231 breast carcinoma models, whereas unencapsulated vicrostatin at the same dosage showed no effect [210]. Furthermore, peptide modification strategies such as cyclization, PEGylation, and conjugation with gold nanoparticles are being developed to shield the peptides from degradation, enhance tissue targeting, and extend circulatory half-life [211]. A notable clinical success involves chlorotoxin (a scorpion peptide), which has been conjugated into nanoparticles and advanced to clinical trials as an imaging and therapeutic agent for glioblastoma, proving that venom-derived macromolecules can successfully navigate regulatory and physiological barriers [212].

## 7. Challenges, Limitations and Future Perspectives of Venom-Derived Anti-Cancer Agents

In spite of the tremendous potential for venom-derived enzyme inhibitors in cancer related ailments, the scientific and translational challenges continue to impede their progression from experimental compounds to clinically approved therapeutics. Many venom peptides, proteins, and enzyme-targeting molecules were found to exhibit remarkable selectivity toward cancer-associated pathways, and their successful therapeutic application requires overcoming substantial limitations related to the following factors mentioned below.

### 7.1. Safety and Off-Target Toxicity

The intrinsic toxicity of venom-derived molecules remains one of the primary barriers to clinical translation. Since venoms have evolved for predation and defense, many of their constituents exhibit potent cytotoxic, neurotoxic, cardiotoxic, or hemo-toxic effects that may damage healthy tissues in addition to cancer cells (Table 7). Furthermore, many venom-derived enzymes inhibitors target highly conserved enzymes or receptors expressed in both normal and malignant cells, increasing the likelihood of off-target toxicity and limiting the therapeutic window. Clinical experience has highlighted these challenges; for example, Ancrod, a thrombin-like serine protease from *Calloselasma rhodostoma*, was discontinued owing to bleeding complications, while the bee venom peptide melittin exhibits potent anti-cancer activity but is restricted by severe hemolysis and systemic toxicity [220]. Future research should therefore focus on rational protein engineering, structure-guided optimization, anti-body-drug conjugates, tumor-activated prodrugs, and ligand-directed nanocarriers to improve tumor selectivity while minimizing systemic toxicity.

### 7.2. Pharmacokinetic Limitations and Drug Delivery

Most venom-derived enzyme inhibitors are peptides or proteins that exhibit poor pharmacokinetic characteristics, including rapid proteolytic degradation, short plasma half-lives, poor oral bioavailability, and rapid renal clearance. These limitations often necessitate parenteral administration and reduce therapeutic efficacy. Moreover, abnormal tumor vasculature, hypoxia, acidic pH, and dense extracellular matrices further restrict drug penetration and accumulation within tumor tissues. Recent advances in PEGylation, peptide cyclization, liposomal formulations, polymeric and lipid nanoparticles, hydrogels, and antibody-mediated delivery have shown considerable promise in improving stability, prolonging circulation time, enhancing tumor targeting, and reducing systemic exposure. Continued optimization of these delivery platforms is expected to significantly improve the clinical performance of venom-derived therapeutics [109,113,114,117,122,123,125,222].

### 7.3. Immunogenicity, Manufacturing and Standardization

As foreign biological macromolecules, venom-derived peptides and proteins may elicit immune responses during repeated administration, resulting in antibody production, hypersensitivity reactions, reduced therapeutic efficacy, and potential anaphylaxis. Simultaneously, large-scale pharmaceutical development is complicated by substantial variability in venom composition arising from differences in species, geographic origin, age, diet, and environmental conditions. The limited availability of natural venom, coupled with the complexity of purification and quality control, further restricts commercial scalability and raises sustainability concerns regarding continued harvesting of venomous species. Recombinant DNA technology, synthetic peptide chemistry, cell-free protein synthesis, and peptide engineering offer promising alternatives for sustainable large-scale production while enabling improved batch consistency and reduced immunogenicity through rational sequence optimization [223,224,225,226].

### 7.4. Limited Mechanistic Understanding and Difficulties in Targeted Delivery

Majority of the venom derived enzyme inhibitors which have been reported for anti-cancer activities in vitro and in vivo often lack the precise molecular mechanisms underlying their effects. These studies are not primarily focused on elucidating downstream signaling pathways, target specificity, structure activity relationships or mechanism of resistance. Cancer is a highly complex disease involving a plethora of factors *viz*., dynamic genetic, epigenetic, and metabolic alterations. Consequently, the venom derived inhibitors may show different responses across the different cancer types or even among patients with the same malignancy. The need of the hour for a rational anti-cancer drug design is deeper understanding about molecular targets, cellular uptake mechanisms, apoptosis pathways, angiogenesis inhibition, immune modulation, and tumor microenvironment interactions. Additionally, the multi-target nature of many venom compounds may complicate mechanistic interpretations and increase the likelihood of unintended biological effects.

Efficient delivery of venom-derived enzyme inhibitors to tumor tissues poses as another major challenge. Systemic administration may result in rapid clearance, non-specific biodistribution, or accumulation in healthy organs. Biological barriers such as renal filtration, enzymatic degradation, and poor tumor penetration can substantially reduce therapeutic efficiency. It is a well-known fact amongst the cancer research community that tumor microenvironment exhibits hypoxia, abnormal vasculature, acidic pH, and elevated interstitial pressure, all of which may limit drug accessibility. On a positive note, enthusiastic research explorations on targeted delivery systems involving nanoparticles, liposomes, dendrimers, hydrogels and ligand-mediated targeting platforms may help in overcoming these limitations [109,113,122,123,124,125].

### 7.5. Insufficient Clinical Translation

Applicability of venom derived inhibitors in cancer therapy is still a very new domain and despite extensive preclinical research, relatively few venom-derived anticancer agents have progressed to clinical trials, and even fewer have achieved regulatory approval. Many promising compounds demonstrate potent activity in cell culture models but fail during animal studies or clinical evaluation due to inadequate pharmacokinetics, or insufficient therapeutic benefit. Moreover, standard preclinical models may not accurately replicate the complexity of human tumors, leading to discrepancies between experimental findings and clinical outcomes. The major setback which every pharmaceutical drug encounter is during the transition from laboratory research to clinical application as it involves huge developmental costs, regulatory complexities and limited commercial investments. Setting aside the financial factors, the issue related to clinical translation can be overcome with the incorporation of advanced models involving patient-derived organoids, 3D tumor cultures, and precision oncology approaches. Although venom-derived peptides have demonstrated encouraging anticancer activity in conventional two-dimensional (2D) cell culture models, these systems often fail to recapitulate the architectural complexity, cellular heterogeneity, and tumor microenvironment observed in vivo. Consequently, patient-derived organoids (PDOs) and three-dimensional (3D) tumor culture models have emerged as more physiologically relevant platforms for preclinical drug evaluation. Several venom-derived compounds, including chlorotoxin [227], disintegrins [228], and melittin [229,230], have shown promising therapeutic effects in 3D tumor spheroids and related advanced culture systems, supporting their translational potential. In contrast, studies evaluating Kunitz-type protease inhibitors in PDOs or patient-derived 3D tumor models remain extremely limited. Most investigations of these inhibitors have been confined to conventional 2D cancer cell lines and xenograft models. Expanding future studies to incorporate PDOs, multicellular tumor spheroids, and organ-on-chip platforms could provide more clinically relevant insights into the efficacy, tissue penetration, mechanism of action, and therapeutic selectivity of Kunitz-type inhibitors, thereby facilitating their translation toward precision oncology.

### 7.6. Ethical, Ecological, and Sustainability Concerns

The last point of contention involving venom derived anti-cancer drugs is the ethical and sustainability concerns. Large-scale harvesting of venoms from snakes, scorpions, spiders, marine organisms, and other venomous species raises ecological and ethical concerns. In the current era of social activism and environmentalism, the industrial investors and governments will be reluctant in contributing towards the development of venom derived inhibitors and the financial constraints involving its development will suffer as a consequence. Now playing the devil’s advocate, we can also not completely ignore the concern of the environmentalists as overexploitation of venomous organisms may threaten the biodiversity and disrupt ecological balance while also exerting tremendous stress on the animals by the venom extraction procedures.

### 7.7. Potential Mechanisms of Tumor Resistance to Venom-Derived Enzyme Inhibitors and Strategies to Overcome Them

Despite increasing evidence supporting the anticancer potential of venom-derived enzyme modulators, the possibility of acquired or intrinsic tumor resistance remains an important translational challenge that has received comparatively limited attention. Although dedicated resistance studies for venom-derived inhibitors remain scarce, established mechanisms of resistance observed for conventional targeted therapies are likely to become relevant as venom-inspired agents advance toward clinical development [231]. One major resistance mechanism involves adaptive target rewiring and compensatory pathway activation. The inhibition of a single enzyme pathway may trigger activation of redundant signaling networks that preserve tumor survival and proliferation. For example, suppression of matrix metalloproteinase activity may induce compensatory upregulation of alternative proteases such as cathepsins or urokinase-associated proteolytic systems, thereby maintaining invasive potential [232]. Tumors may additionally develop resistance through altered target expression or structural modification. Reduced expression of membrane-associated targets, mutations affecting inhibitor-binding interfaces, or altered enzyme localization may diminish sensitivity to venom-derived compounds. Similar adaptive responses have been reported for kinase inhibitors and other targeted anticancer agents [233]. Another important mechanism involves drug transport and intracellular availability. An increased expression of ATP-dependent efflux transporters and altered membrane composition may reduce intracellular accumulation of venom-derived peptides and protein-based inhibitors. Furthermore, rapid proteolytic degradation within the tumor microenvironment can reduce biological exposure and shorten therapeutic duration [234]. The tumor microenvironment itself may also contribute to resistance through hypoxia, stromal remodeling, immune suppression, and metabolic adaptation, all of which can alter responsiveness to enzyme-targeted interventions. These factors may be particularly relevant for venom-derived compounds whose activity depends on extracellular accessibility or receptor engagement [235].

Several strategies may help mitigate resistance and improve translational potential. These include combination approaches integrating venom-derived inhibitors with chemotherapy, immunotherapy, or pathway co-inhibition; structure-guided optimization to enhance target affinity and reduce degradation; development of multifunctional or multi-target venom-inspired constructs; and advanced delivery technologies including nanoparticles, conjugates, and controlled-release systems [12]. Longitudinal molecular profiling and biomarker-guided patient stratification may further improve durability of therapeutic responses. Understanding resistance biology should become an integral component of future venom-derived anticancer research, shifting development efforts from proof-of-concept cytotoxicity toward durable mechanism-based therapeutic design.

## 8. Conclusions

Animal venoms represent a structurally diverse source of bioactive molecules with emerging relevance for anticancer drug discovery. This review demonstrates that the therapeutic value of venom-derived compounds extends beyond direct cytotoxicity and includes modulation of enzyme-regulated pathways involved in extracellular matrix remodeling, angiogenesis, tumor invasion, metabolic adaptation, and apoptotic signaling. Current evidence most strongly supports venom-derived targeting of proteolytic and extracellular signaling systems, particularly matrix metalloproteinases, phospholipases A_2_, serine protease-associated pathways, and redox-regulated mechanisms. Representative venom-derived scaffolds including disintegrins, phospholipase-associated peptides, Kunitz-type inhibitors, chlorotoxin-derived systems, and L-amino acid oxidase-related platforms have shown encouraging preclinical activity through suppression of tumor dissemination and modulation of tumor–microenvironment interactions. However, the level of evidence differs considerably across enzyme classes. In contrast to protease-related targets, direct venom-derived inhibition of topoisomerases, epigenetic enzymes, and nucleotide biosynthesis enzymes remains insufficiently validated and currently represents an important knowledge gap rather than an established therapeutic direction. A major conclusion emerging from this review is that future progress will depend less on continued discovery of new venom components and more on optimization, validation, and translation of existing molecular scaffolds. Several challenges continue to limit clinical advancement, including insufficient selectivity, rapid degradation, immunogenicity, pharmacokinetic constraints, manufacturing scalability, variability in venom composition, and limited clinical evidence. These limitations should be recognized when interpreting current preclinical findings.

Future development of venom-derived enzyme inhibitors will likely require integrated strategies combining peptide engineering, structure–activity relationship optimization, computational venomics, molecular modeling, advanced delivery systems, and standardized mechanistic validation. Approaches integrating synthetic analog development, targeted delivery technologies, and multi-omics-guided candidate prioritization may improve translational success. Hence, venom-derived enzyme modulators should presently be regarded not as near-clinical replacements for existing anticancer therapies but as a rapidly evolving source of mechanistically novel molecular templates. Continued integration of venomics, medicinal chemistry, and translational oncology will determine whether these evolutionarily refined molecules can ultimately be converted into clinically actionable anticancer therapeutics.

## Figures and Tables

**Table 2:** Major enzyme targets in cancer and representative venom-derived inhibitors/mechanisms.

Enzyme Class	Role in Cancer	Clinical Inhibitors	Venom-Derived Example	Evidence Status	Remarks	References
Matrix Metalloproteinases (MMPs)	ECM remodeling; invasion	MMP inhibitors	BJ46A; venom peptide libraries	Established	MMP suppression; anti-angiogenesis	[12,40,41,42]
Serine Proteases (uPA, matriptase)	Proteolysis; metastasis	uPA inhibitors	PIMR, PIVL	Established	Protease inhibition	[43,44,45]
Cathepsins	ECM degradation; EMT	Cathepsin inhibitors	-	Gap Identified	No validated venom inhibitor	[46,47]
APN/CD13	Angiogenesis; metastasis	Bestatin	-	Gap Identified	No validated venom inhibitor	[48,49,50]
Kinases	Growth signaling	EGFR, VEGFR, PI3K inhibitors	Disintegrins; venom peptides	Emerging	Signaling modulation	[31,51,52]
Topoisomerases	DNA replication	Irinotecan, etoposide	-	Gap Identified	No validated selective inhibitor	[53,54,55,56]
PLA_2_	Membrane signaling	Secreted PLA_2_ inhibitors	Snake PLA_2_; bee PLA_2_	Established	Membrane stress; apoptosis	[57,58,59,60,61]
HDACs	Epigenetic regulation	Vorinostat, panobinostat	-	Gap Identified	Indirect epigenetic effects	[62,63,64]
DNMTs	DNA methylation	Azacitidine, decitabine.	-	Gap Identified	No direct evidence	[65,66]
Ribonucleotide reductase	DNA synthesis	Gemcitabine	-	Gap Identified	No validated inhibitor	[67,68]
Thymidylate Synthase	dTMP synthesis	5-FU, raltitrexed	-	Gap Identified	No validated inhibitor	[69]

**Table 3:** Integrated SAR–cancer application relationship [6,9,17].

Structural Feature	Mechanism	Cancer Application
Disulfide bonds	Structural stability	Target specificity
RGD/KGD motifs	Integrin binding	Anti-metastatic, anti-angiogenic
Catalytic domains	ECM degradation/modulation	Metastasis control
Amphipathic structure	Membrane disruption	Direct cytotoxicity
Ion channel targeting	Signal modulation	Growth inhibition
Amphipathic helix	Membrane disruption	Direct tumor killing
Positive charge	Tumor selectivity	Targeted cytotoxicity
Hydrophobic domain	Membrane insertion	Pore formation
Flexible structure	Broad interaction	Multi-target action
Nanocarrier modification	Reduced toxicity	Safe drug delivery

**Table 4:** Selected venom-derived toxins relevant to enzyme inhibition, their biological functions, and experimental materials.

Toxin/Peptide	Source Organism	Description/Function	Experimental Material	UniProtKB	Primary Target(s)	Reference(s)
Melittin	*Apis mellifera* (Bee venom)	Membrane-active peptide inducing apoptosis	Purified pep-tide	P01501	PLA_2_, membranes	[128]
PLA_2_	*Bothrops jararacussu* (Snake Venom)	Hydrolyses phospholipids, inflammatory mediator	Purified enzyme	P20474	Membrane phospholipids	[129]
Chlorotoxin	*Leiurus quinquestriatus* (Scorpion venom)	Tumor-targeting peptide inhibiting invasion	Purified pep-tide	P45639	MMP-2, Cl^−^ channels	[130]
ω-Conotoxin MVIIA	*Conus magus* (Cone Snail)	Blocks voltage-gated Ca^2+^ channels	Sequence-defined peptide	P05484	Ca^2+^ channels	[131]
Batroxobin	*Bothrops atrox* (Snake Venom)	Thrombin-like serine protease	Purified enzyme	P04971	Fibrinogen	[132]
L-amino acid oxidase	*Naja naja* (Snake Venom)	Induces apoptosis via ROS	Purified enzyme	Not re-ported	Oxidative pathways	[133]
Polybia-MP1	*Polybia paulista* (Wasp Venom)	Cytoplasm leakage and cell death	Synthetic peptide	P0C1Q4	PC-3 cell line	[33]
Crotoxin	*Crotalus durissus* (Snake venom)	Neurotoxic PLA_2_ complex	Purified toxin complex	P62202 P08878	PLA_2_, synapses	[134]
Contortrostatin	*Agkistrodon contortrix* (Snake venom)	Anti-angiogenic disintegrin	Purified dis-integrin	Q9IAB0	Integrins	[135]
Cytotoxin 3	*Naja atra* (Snake venom)	Membrane-disrupting cardiotoxin	Purified cytotoxin	P01447	Membranes	[136]

**Table 5:** Selected computational and translational insights into venom-derived enzyme inhibitors.

Toxin	Target	Disease Context	Computational Methods	Key Findings	Clinical Status	Ref.
Melittin	Membranes/PLA_2_	Breast cancer	Docking + MD (ClusPro 2.0 and Desmond software)	Good predicted value of 0.86 in *E. coli*, stable docked complex.	Preclinical	[143]
Chlorotoxin	MMP-2	Glioblastoma	Docking (HPEPDOCK, HADDOCK, AlphaFold2 with a local copy of ColabFold) + MD (GROMACS2022)	Identified a pathway between CTX and several protein targets that GBM cells express.	--	[144]
ω-Conotoxin MVIIA	Ca^2+^ channel	Pain	MD + docking	Stable channel binding.	FDA approved	[145]
Batroxobin	Fibrinogen	Thrombosis	Docking	Substrate specificity.	Clinical use	[146]
*Lansbermin-I*	integrins	Glioblastoma	Docking	Selective prototype for Glioblastoma therapy.	Preclinical	[147]

**Table 6:** Summary of representative in vivo studies evaluating venom-derived anticancer compounds against tumor growth, metastasis, angiogenesis, and targeted delivery.

Venom-Derived Compound	Venom Source	Animal Model	Dose/Administration	Principal Molecular Target/Mechanism	Major In Vivo Outcome	Reference(s)
NnV (crude venom)	*Nemopilema nomurai* (jellyfish)	HepG2 xenograft mouse model	-	Inhibits TGF-β-induced EMT via Smad3/Smad4 and NF-κB signaling	Reduced tumor dissemination and suppressed metastasis.	[191]
DisBa-01	*Bothrops alternatus*	B16F10 melanoma-bearing mice; Matrigel angiogenesis model	0.05–4 mg/kg (i.v.); 0–1000 nM (Matrigel plug)	αvβ3 integrin antagonist; inhibits angiogenesis and tumor cell adhesion	Reduced angiogenesis and pulmonary metastasis.	[205]
Flavoridin (FL) and Kistrin (KR)	Snake venom disintegrins	B16F10 lung metastasis mouse model	Intravenous administration (study protocol)	Inhibits integrin-mediated tumor cell adhesion	Significantly reduced lung metastatic burden.	[206]
Crotamine	*Crotalus durissus terrificus*	B16F10 melanoma-bearing mice	Daily administration	Selectively accumulates in proliferating tumor cells	Reduced tumour growth and prolonged survival.	[207]
Lycosin-I-functionalized gold nanoparticles/nanorods (LGNPs/LGNRs)	*Lycosa singoriensis* (wolf spider)	HeLa xenograft mouse model	10 mg/kg (i.v.); NIR (808 nm) irradiation for LGNRs	Tumor-selective cell-penetrating peptide enabling targeted nanoparticle delivery and photothermal therapy	Enhanced tumour targeting and effective tumour ablation.	[208]

**Table 7:** Selected venom-derived compounds whose clinical development was discontinued or limited due to toxicity and safety concerns [220,221].

Compound	Venom Source	Intended Indication	Toxicity Issue	Development Outcome
Ximelagatran (venom-inspired, not directly isolated)	Based on peptides from snake venom thrombin inhibitors	Anticoagulation	Severe hepatotoxicity (elevated liver enzymes and liver injury)	Withdrawn from the market
Ancrod	Malayan pit viper	Acute ischemic stroke	Increased risk of intracranial hemorrhage and bleeding complications	Clinical development discontinued after Phase III
Batroxobin	Bothrops atrox and related pit vipers	Thrombotic disorders	Bleeding risk and inconsistent clinical benefit limited broader approval	Limited regional use; not widely approved internationally
Cobrotoxin	Chinese cobra	Chronic pain and cancer pain	Neuromuscular toxicity, respiratory depression, and narrow therapeutic index	Clinical development remained limited
Contulakin-G	*Conus geographus*	Chronic pain	Sedation, dizziness, and dose-limiting central nervous system adverse effects	Development discontinued
Chlorotoxin (early therapeutic conjugates)	Deathstalker	Brain tumors	Some conjugated formulations encountered off-target toxicity and insufficient safety margins, prompting redesign rather than complete abandonment	Several early candidates discontinued; newer derivatives continue to be investigated

## Data Availability

The original contributions presented in this study are included in the article. Further inquiries can be directed to the corresponding author.
